# Mobile genetic elements encoding antibiotic resistance genes and virulence genes in *Klebsiella pneumoniae*: important pathways for the acquisition of virulence and resistance

**DOI:** 10.3389/fmicb.2025.1529157

**Published:** 2025-02-24

**Authors:** Bin Han, Chunlin Feng, Yuan Jiang, Caihong Ye, Yueshuai Wei, Jinbo Liu, Zhangrui Zeng

**Affiliations:** ^1^Department of Laboratory Medicine, The Affiliated Hospital of Southwest Medical University, Luzhou, China; ^2^Sichuan Province Engineering Technology Research Center of Clinical Diseases Molecular Diagnosis, Luzhou, China; ^3^Molecular Diagnosis of Clinical Diseases Key Laboratory of Luzhou, Luzhou, China; ^4^Department of Laboratory Medicine, Southwest Medical University, Luzhou, China

**Keywords:** mobile genetic elements, antibiotic resistance genes, virulence genes, extracellular vesicles, *K. pneumoniae*

## Abstract

*Klebsiella pneumoniae* is an opportunistic pathogen primarily associated with nosocomial infections, characterized by a propensity for multi-drug resistance and the potential evolution into hypervirulent strains. Based on its phenotypic and genotypic characteristics, *K. pneumoniae* can be classified into two types: classical *K. pneumoniae* (cKP) and hypervirulent *K. pneumoniae* (hvKP). The spread of mobile genetic elements (MGEs) in *K. pneumoniae* has led to the emergence of carbapenem-resistant *K. pneumoniae* (CRKP) and carbapenem-resistant hypervirulent *K. pneumoniae* (CR-hvKP). The emergence of CR-hvKP is particularly concerning due to its multidrug resistance, high pathogenicity, and increased transmissibility. This review summarizes the types of MGEs present in *K. pneumoniae*, the mechanisms of horizontal gene transfer (HGT) mediated by these mobile elements, their roles in the dissemination of antibiotic resistance genes (ARGs) and virulence genes, and the relationships among MGEs that resemble Russian dolls or exhibit hybrid characteristics. Additionally, the clinical treatment and epidemiological characteristics of CR-hvKP are discussed. Given the high variability and transmissibility of MGEs, continuous monitoring and control of the variation and transmission of such genetic material in *K. pneumoniae* should be prioritized.

## 1 Introduction

*K. pneumoniae* is a well-known opportunistic and hospital-acquired pathogen capable of causing both pulmonary and non-pulmonary infections. These infections can be invasive, including liver abscesses, endophthalmitis, and meningitis (Chew et al., 2017). *K. pneumoniae* strains can generally be classified into cKPs and hvKPs based on their disease profiles and genetic characteristics (Zou and Li, 2021). The pathogenicity of *K. pneumoniae* is closely linked to various virulence factors, including the capsule, which resists neutrophil phagocytosis and serum complement-mediated sterilization (Cortés et al., 2002; Ares et al., 2019); Lipopolysaccharide (LPS), a type of endotoxin that resists immune phagocytosis and induces fever (Papo and Shai, 2005); type I and III fimbriae, which enable adherence to host cells and facilitate infection (Schroll et al., 2010; Wu et al., 2012); and siderophores or iron acquisition systems (Martin and Bachman, 2018). HvKP is responsible for community-acquired infections that predominantly affect young and adult hosts, such as pyogenic liver abscesses, endophthalmitis, and meningitis, but it has historically been susceptible to antibiotics. CRKP is commonly associated with hospital-acquired urinary tract infections, pneumonia, sepsis, and soft tissue infections. The outbreaks and rapid spread of CRKP in hospitals have become a major public health challenge due to the lack of effective antimicrobial treatments. HvKP and CRKP first emerged as distinct lineages during the early stages of *K. pneumoniae* evolution. However, with the widespread dissemination of MGEs in *K. pneumoniae*, the current boundary that separates these two pathotypes is diminishing. CR-hvKP can arise when hvKP or CRKP acquires plasmids carrying carbapenem resistance genes or virulence genes, or when cKP acquires hybrid plasmids containing both types of genes. CR-hvKP has been widely reported in Asia, particularly in China, due to its multidrug resistance, high virulence, and contagious nature, which poses a significant threat to clinical treatment.

MGEs play a critical role in both antibiotic resistance and pathogenicity in CR-hvKP. Virulence and resistance genes are typically located within MGEs and are horizontally transferred among bacteria. MGEs are widely distributed throughout the bacterial genome and facilitate the intra-or inter-bacterial transfer of virulence and resistance genes. The primary mechanisms of HGT are transformation, conjugation, and transduction in prokaryotes (Li P. et al., 2022). MGEs are essential for maintaining bacterial genome stability, enhancing environmental adaptation, and increasing gene diversity through the functional genes they carry (Frost et al., 2005).

Plasmids are primary MGEs that facilitate the dissemination of ARGs, virulence genes, and other functional genes in *K. pneumoniae*. Plasmids are categorized as conjugative or mobilizable, depending on their ability to self-transfer. However, this does not imply that all plasmids are capable of conjugation. Furthermore, recent studies have underscored the importance of extracellular vehicles (EVs) in mediating the transfer of these functional genes (Mathieu et al., 2019).

Research on resistance genes, virulence genes, and associated MGEs is crucial for mitigating the global health threat posed by *K. pneumoniae*. This review is organized around the concept of MGEs and is divided into four sections. The first section provides an overview of the primary types of MGEs found in *K. pneumoniae* and their roles in the transmission of resistance and virulence genes. The second section examines the mechanisms of HGT involving MGEs, including transformation, transduction, conjugation, and vesiduction. The third section explores the interrelationships between MGEs, drawing analogies to Russian nesting dolls or intersections. Finally, the fourth section discusses the impact of MGEs on the clinical management of *K. pneumoniae*.

## 2 Types of MGEs

**Figure 1:**
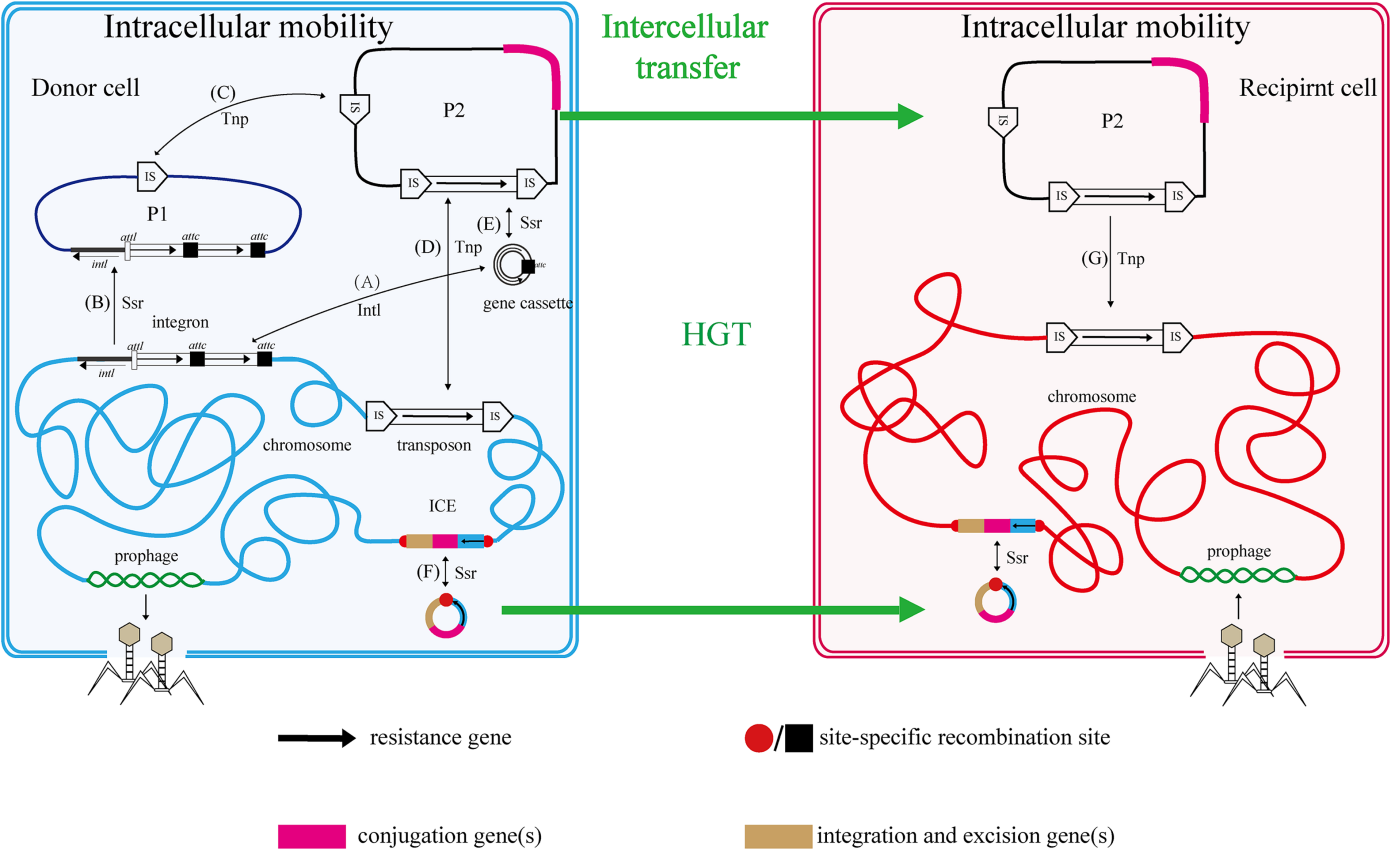
Relationship and mobility of MGEs. Two cells, one donor (blue envelope and chromosome with plasmids P1 and P2) and one recipient (red), are shown with various mobile genetic elements (MGEs). The functions of genes and resistance genes they carry are indicated by color coding and fat black arrows, respectively. Thin black arrows indicate intracellular processes, with those mediated by an integrase protein labeled Intl, a transposase protein labeled Tnp and those mediated by a site-specific recombinase protein labeled Ssr. Thick green arrows represent HGT. Capital letters in parentheses represent the process of intracellular MGEs transfer in cells. Refer to the text for more details.

The mobile genome of bacteria encompasses all MGEs within a bacterial genome, including gene cassettes, integrons, plasmids, transposable elements, insertion sequences (IS), transposons (Tn), prophages, and integrative and conjugative elements (ICE), among others (Figure 1).

### 2.1 Gene cassettes and integrons

A gene cassette, a small MGE, consists of multiple genes and recombination sites and can be captured by integrons (Partridge et al., 2009; Xu et al., 2018). Currently, the genes within gene cassettes are primarily associated with drug resistance traits. Integrons serve as assembly platforms that capture gene cassettes and incorporate exogenous genes through site-specific recombination, allowing for the internalization and expression of these foreign genes (Hall and Collis, 1995; Mazel, 2006; Escudero et al., 2015). Although integrases cannot excise it from the chromosome, rendering the integron itself immobile, they can depend on recombinases or transposases for genome-wide migration.

### 2.2 Transposable elements

Transposon elements, including insertion sequences (ISs) and transposons (Tns), are MGEs that mediate intracellular gene transfer (Partridge et al., 2018). ISs and Tns are discrete, mobile DNA segments that can relocate within the genome through a series of processes, including excision and reintegration.

#### 2.2.1 Insertion sequence

Insertion sequences, which are essentially short DNA sequences, represent the smallest and most abundant autonomous transposable elements (Siguier et al., 2015). They play a crucial role in the evolution of the host genome, contributing to gene sequestration, transmission, mutation, and activation, as well as facilitating plasmid and chromosome rearrangements. In recent years, several transposable elements (TEs) closely related to known insertion sequences (IS) have been identified, referred to as transporter IS (tIS). However, these tIS carry passenger genes that are not directly involved in transposition (Siguier et al., 2009). Passenger genes include transcription regulators (e.g., ISNha5, a member of the IS1595 family), methyltransferases (e.g., IS220, IS1380 family), and antibiotic resistance genes (e.g., ISCgl1, IS481 family). IS that mediate the movement of drug-resistant genes have been documented in *K. pneumoniae* (Cienfuegos-Gallet et al., 2017; Yang Y. et al., 2021; Pu et al., 2023b; Li et al., 2024). The addition or deletion of insertion sequences in plasmids may serve as a potential mechanism for mediating their evolution or rearrangement. For instance, plasmid Eco-N-1-p results from the deletion of the blaOXA-carrying IS26 from plasmid GX34p4_OXA-181 and the addition of IS26 from the chromosome that carries blaSHV, along with an IS5-like sequence from the chromosome that “hijacks” the blaNDM located in other plasmids (Zhang et al., 2023). These plasmids can be transferred into *K. pneumoniae*. The carbapenem-resistant plasmid pK186_KP is derived from the cointegration of the IncN and IncFII plasmids, an event that is entirely dependent on the transposition of IS26 (Chen Q. et al., 2022). Additionally, IS-mediated transposition in the mgrB gene may explain *K. pneumoniae*’s resistance to colistin (Aris et al., 2020; Yan et al., 2021; Sánchez-León et al., 2023).

#### 2.2.2 Transposons

Tns and ISs function similarly in bacterial genomes by facilitating genetic mobility. Composite transposons carry specific genes, such as ARGs (Mansour et al., 2017; Zhou et al., 2017; Abril et al., 2021; Tian et al., 2022; Shi et al., 2024), flanked by identical or highly homologous ISs. In contrast, unit transposons are larger elements than ISs, consisting of a transposase gene and an internal “passenger” gene, which may encode antibiotic resistance. They are flanked by inverted repeats (IRs) rather than a pair of ISs. The beta-lactamase gene blaKPC-2 in a *K. pneumoniae* isolate from the United States was found on a novel transposon called Tn4401 (Naas et al., 2008). This transposon was 10 kb long and contained two 39-bp imperfect inverted repeat sequences. In addition to the blaKPC-2 gene, Tn4401 also carried a transposase gene (Garbari et al., 2015; Yoon et al., 2018), a resolvase gene, and two novel insertion sequences, ISKpn6 and ISKpn7. In Switzerland, a Tn3-like transposon harboring blaVIM-1 was identified in a new plasmid (pOW16C2) from a *K. pneumoniae* strain isolated from river water (Zurfluh et al., 2015). The blaOXA-48 gene in plasmid pOXA-48a, isolated from *K. pneumoniae*, had been integrated through the acquisition of the Tn1999 composite transposon (Poirel et al., 2012; Li W. et al., 2022). The dissemination of the Tn125 transposon facilitated the spread of the blaNDM gene in Enterobacteriaceae, Acetobacteriaceae, and Pseudomonadaceae (Bonnin et al., 2014; Li et al., 2020).

### 2.3 Prophages

Phages can be classified into two types, virulent and temperate phages, based on their distinct life cycles within the host (Casjens, 2003). Virulent phages follow a classical cycle consisting of five stages: adsorption, invasion, proliferation, assembly, and lysis, ultimately leading to the release of progeny phages. In contrast, temperate phages integrate their genetic material into the host genome and can adopt a lysogenic state. Under specific inducing conditions, temperate phages can enter the lytic phase, resulting in the lysis of the host bacterium. Prophages are the nucleic acids of temperate phages that integrate into the host genome at specific sites, allowing them to replicate alongside the host chromosomes (Canchaya et al., 2003). HGT occurs through the imprecise excision of the prophage, which can carry host genes, leading to cell lysis under induced conditions and subsequent infection of a new host. Prophage-mediated HGT is facilitated by transduction, which includes specific transduction, generalized transduction, and lateral transduction (as described in the section on transduction below). Multiple prophages have been identified on the chromosomes and plasmids of *K. pneumoniae*, exhibiting diversity and widespread dissemination (Gabashvili et al., 2020; Shen et al., 2020; Kang et al., 2023). Notably, a prophage containing the blaKPC gene has been discovered in the chromosomes of KPC-producing *K. pneumoniae* strains (Chen et al., 2015).

### 2.4 Plasmid

*K. pneumoniae*, which mediates the transfer of resistance genes, is primarily associated with various classes of antibiotics to which it develops resistance. These include tetracyclines (tet), sulfonamides (sul), β-lactams (bla), aminoglycosides (AAC), mucormycetes (MCR), quinolones (QNR), and multidrug-resistant bacteria (MDR). Information regarding these plasmids, as collected from the literature, is presented in Table 1. This table was utilized to create plasmid maps that illustrate the relevant resistance gene information in *K. pneumoniae*. Furthermore, plasmids also facilitate the transfer of genes that encode functions enabling recipient cells to better adapt and survive in their environments (Bellanger et al., 2014). To better understand the contribution of plasmids in HGT, it is important to distinguish between transmissible and non-transmissible plasmids. Transmissible plasmids include conjugative plasmids and mobilizable plasmids (Fang and Zhou, 2020). Specifically, conjugative plasmids carry all the essential genes for self-transmission through conjugation, including a Type IV secretion system (T4SS), a T4SS coupling protein (T4CP), relaxasome accessory factors (RAFs), and a relaxase gene (Ramsay and Firth, 2017). Essential genes are shown on the *K. pneumoniae* plasmid map in the following description.

**Table 1:** GenBank accession numbers.

Plasmid name	Inc group	Conjugative	GenBank	NCBI reference sequence
pKpQIL	FII	Conjugative	GU595196.1	NC_014016.1
pKpn1693-CTXM	FII	—	CP047597.1	NZ_CP047597.1
pKP91	FII	Conjugative	MG736312.1	NZ_MG736312.1
p0716-KPC	FII	Conjugative	KY270849.1	NZ_KY270849.1
p12181-KPC	FII	Non-conjugative	KY270850.1	NZ_KY270850.1
pKpn-431cz	FII	Conjugative	KY020154.1	NZ_KY020154.1
pIMP1572	FII_K_	Conjugative	MH464586.1	NZ_MH464586.1
p1220-CTXM	FII_K_	Conjugative	KY174332.1	NZ_KY174332.1
pBK32179	FII_K_	Conjugative	JX430448	NC_020132.1
pKPN3	FII_K_	—	CP000648.1	NC_009649.1
pKPN4	FII_K_	—	CP000649.1	NC_009650.1
pKPX-1	FIIA	Non-conjugative	AP012055.1	NC_021198.1
pKPX-2	F	Conjugative	AP012056.1	NC_021199.1
pKP048	F	Conjugative	FJ628167.2	NC_014312.1
pBK30661	FIA	Non-conjugative	KF954759.1	NC_025185.1
pBK30683	FIA	Conjugative	KF954760.1	NC_025131.1
pA1705-qnrS	FIB	Non-conjugative	MG764551	—
p911021-tetA	FIB	Non-conjugative	MG288679	—
p1642-2	FIB	Non-conjugative	MF156696	NZ_MF156696.1
pKAM260_1	FIB	—	AP023265	—
pIMP-HZ1	N	Conjugative	JX457479	—
pNDM-BTR	N	Conjugative	KF534788.2	NC_022375.2
pFCF1305	N	Conjugative	CP004366.2	NC_021664.2
pFCF3SP	N	Conjugative	CP004367.2	NC_021660.2
pKPI-6	N	Conjugative	AB616660.2	—
pBK31551	N	Conjugative	JX193301	NC_019888.1
pNL194	N	Conjugative	GU585907	NC_014368.1
pOW16C2	N	Conjugative	KF977034	NC_025186.1
pK186_KPC	N	Conjugative	CP076521	NZ_CP076521.1
pNDM-HN380	X	Conjugative	JX104760.1	NC_019162.1
pKpS90	X	Conjugative	JX461340	NC_019384.1
pBK31567	X	Conjugative	JX193302	NC_019899.1
pIncX-SHV	X	Conjugative	JN247852	NC_019157.1
pKPC-NY79	X	Conjugative	JX104759	NC_019161.1
pIncAC-KP4898	A/C	Conjugative	KY882285	—
pKP-Gr642	A/C	Conjugative	KR559888	NZ_KR559888.1
pKP-Gr8143	A/C	Non-conjugative	KR559889	NZ_KR559889.1
pIMP-PH114	A/C	Conjugative	KF250428	NC_022652.1
pKPoxa-48 N1	L/M	Mobilizable	KC757416	NC_021488.1
pEGY22_CTX-M-14	L/M	—	ON261190	NZ_ON261190.1
pE71T	L/M	Conjugative	KC335143	NC_023027.1
pFOX-7a	L/M	Conjugative	HG934082.1	NC_025134.1
pTMTA63632	L/M	Conjugative	AP019667	NZ_AP019667.1
pYDC676	R	—	KT225462	NZ_KT225462.1
pKPS30	R	—	KF793937	NC_023314.1
pKPS77	R	—	KF954150	NC_023330.1
pKP1780	R	—	JX424614	NC_021576.1
pKPC-LK30	R	—	KC405622	NC_020893.1
pR50-74	R	—	CP040363	NZ_CP040362.1
pKPN535a	Q	Mobilizable	MH595533.1	NZ_MH595533.1
p60136	Q	Mobilizable	KP689347	NZ_KP689347.1
pMCR_KP1511	P	Conjugative	KX377410	NZ_KX377410.1
pNDM-MAR	H	—	JN420336	NC_016980.1
p51015_NDM-1	H	—	CP050380.1	NZ_CP050380.1

#### 2.4.1 Resistance plasmid

The dissemination of antimicrobial resistance in *K. pneumoniae* is primarily associated with the exchange of DNA both inter-and intra-specifically, particularly through the horizontal transfer of plasmid-located resistance genes. These plasmids can confer resistance to a wide range of antimicrobial classes, including β-lactams, aminoglycosides, chloramphenicol, macrolides, sulfonamides, trimethoprim, tetracyclines, and quinolones (Carattoli, 2009). Based on plasmid incompatibility, drug-resistant plasmids in *K. pneumoniae* can be categorized into several groups, including IncF, IncN, IncX, IncA/C, IncL/M, IncR, IncP, IncH, IncI, and IncW. Plasmids within the same incompatibility (Inc) group are characterized by shared elements of their replication or partition systems, which prevent their stable coexistence within the same cell (Novick, 1987). The major plasmid incompatibility groups associated with drug resistance genes in *K. pneumoniae* include IncF, IncN, IncA/C, IncL/M, and IncX. (Plasmid group maps should be inserted separately into the appropriate sections of the article; however, IncR, IncQ, IncP, and IncH will be included in the other incompatibility groups).

##### 2.4.1.1 IncF

**Figure 2:**
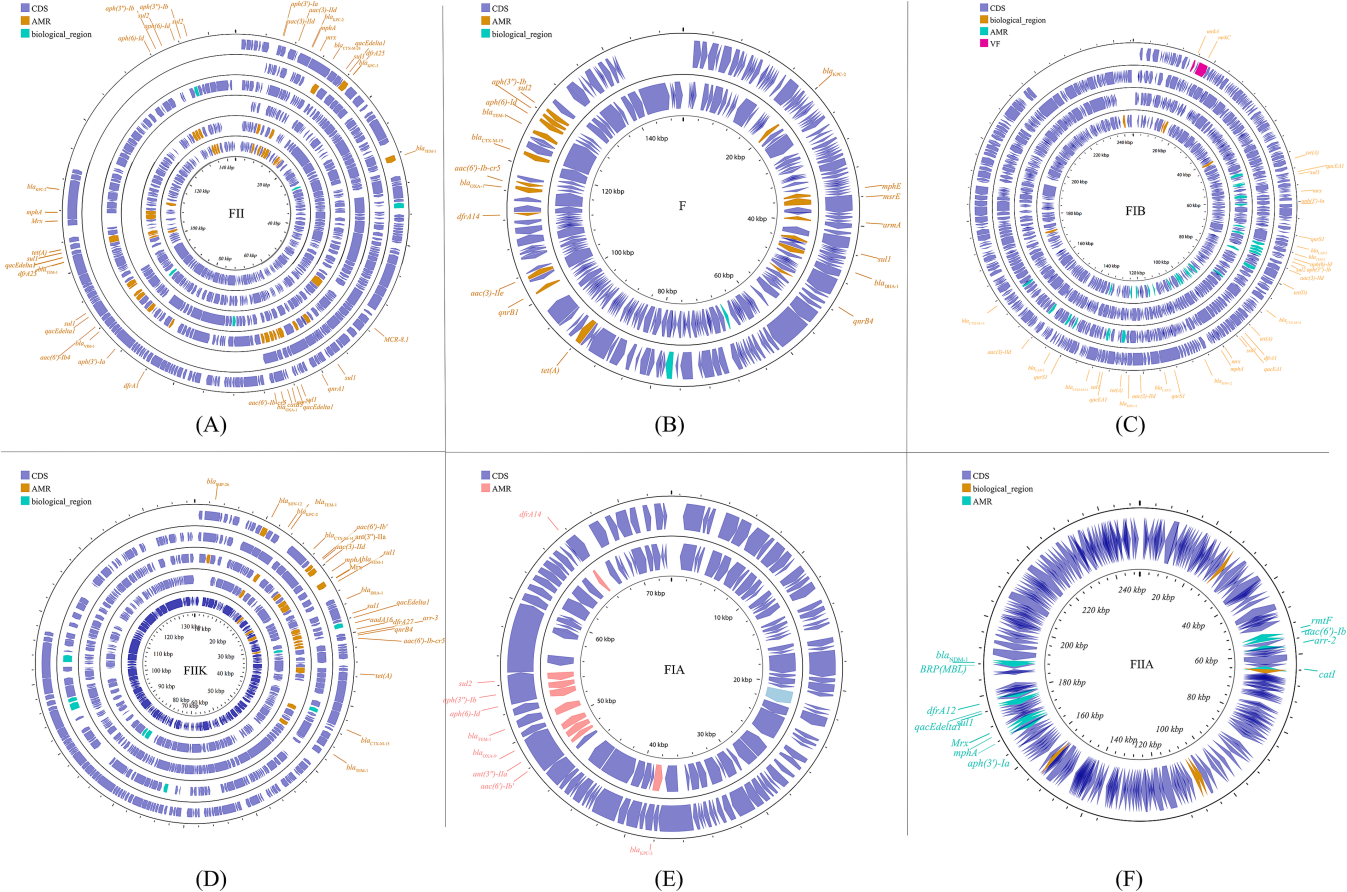
The IncF plasmid group contains multiple subfamilies, which in this paper include incFIA, incFIB, incFII, incFIIA, and incFIIK. again, for the specific plasmid names in the different subtypes are listed behind: FII **(A)**: p0716-KPC, p12181-KPC, pKP91, pKpn-431cz, pKpn1693-CTXM, pKpQIL; F **(B)**: pKP048, pKPX-2; FIB **(C)**: p1642-tetA, p911021-tetA, pA1705-qnrS, pKAM260_1; FIIK **(D)**: p1220-CTXM, pBK32179, pIMP1572, pKPN3, pKPN4; FIA **(E)**: pBK30661, pBK30683; FIIA **(F)**: pKPX-1. Plasmids of the same plasmid group were placed in the same plasmid profile, and we marked their resistance genes and virulence genes in the plasmid profile. The sequence of plasmids in the plasmid profile from inside to outside is **(A)**: p0716-kpc, p12181-kpc, pkp91, pkpn-431cz, pkpn1693-ctxm, pkpqil; **(B)**: pKP048, pKPX-2; **(C)**: p1642-tetA, p911021-tetA, pA1705-qnrS, pKAM260_1; **(D)**: p1220-CTXM, pBK32179, pIMP1572, pKPN3, pKPN4; **(E)**: pBK30661, pBK30683; **(F)**: pKPX-1. CDS, coding DNA sequence; AMR, antimicrobial resistance; VF, virulence factor.

The predominant incompatibility group among Enterobacteriaceae is the IncF plasmids, which have been described globally (Carattoli, 2009; Mathers et al., 2015). The prevalent resistance genes found on IncF plasmids include extended-spectrum β-lactamases (ESBLs) (Shen et al., 2008), carbapenemase-encoding genes, aminoglycoside-modifying enzyme genes, and plasmid-mediated quinolone resistance (PMQR) genes. However, IncF plasmids are typically low-copy-number plasmids, >100 kb in size, and often carry more than one replicon that promotes the initiation of replication, relying on both self-encoded and host-encoded factors for replication (Villa et al., 2010; Ogbolu et al., 2013). IncF plasmids are primarily associated with ESBLs, particularly *blaCTX-M* and *blaTEM*. Additionally, IncF plasmids have been implicated in the transmission of carbapenemase genes, specifically *blaKPC* and *blaNDM*, within the Enterobacteriaceae family (e.g., *K. pneumoniae* and *Escherichia coli*). In many countries, the IncF group dominates the spread of KPC-2 and KPC-3. IncF plasmids facilitate the dissemination of resistance genes by encoding regions essential for conjugative transfer, replication, and segregational stability in *K. pneumoniae* and other species. This group has been reported in numerous studies focusing on KPC-, NDM-, and OXA-producing *K. pneumoniae* across different countries. By searching for the keyword and limiting the scope to *K. pneumoniae*, we collected information on 20 specific reported plasmids (Figure 2; Supplementary Figure S1).

PKpQIL was the first documented occurrence of an IncF plasmid harboring blaKPC in *K. pneumoniae* ST258 (Leavitt et al., 2007). Plasmid pKpQIL is a multi-replicon, self-transferable plasmid with a size of 113,637 bp and belongs to the IncFII group. It contains numerous drug resistance and mercury resistance genes, including *blaKPC-2/-3*, *blaNDM-1*, *blaSHV-11*, *blaTEM-1*, *ΔblaOXA-9*, *ΔaadA1*, *merA*, *merC*, *merD*, and *merE* (Leavitt et al., 2010; Hammad et al., 2023). Subsequently, new variants of pKpQIL have emerged globally, including pKpQIL isolated in Egypt showing its role in the transmission of PMQRs and *blaNDM* (Hammad et al., 2023); pKpQIL-IT, isolated in Italy, which contains *blaKPC-3*, *blaTEM-1*, and *blaSHV-11* genes and exhibits resistance to kanamycin (García-Fernández et al., 2012); and several isolates from hospitals in New Jersey and New York City, including pKpQIL, -03, -04, -10, -234, -Ec, and -Ea, all of which exhibited comparable profiles of antimicrobial and mercury resistance genes, such as *blaKPC-2/-3*, *blaTEM-1*, *blaOXA-9*, *aadA1*, *merA*, *merC*, *merD*, and *merE* (Chen et al., 2014). Examples of IncF plasmids in *K. pneumoniae* carrying *the blaNDM* gene include pKPX-1 (250,444 bp) and pKPX-2 (250,444 bp), which were isolated from a Taiwanese patient in New Delhi (Huang et al., 2013). The former is equipped with β-lactam resistance (*blaNDM-1*) and aminoglycoside resistance genes (*rmt*, *aac (69)-Ib*, *aph(39)-I*, and *aadA2*), while the latter exhibits β-lactam resistance (*blaCTX-M-15*, *blaNDM-1*, *blaTEM-1*, and *blaOXA-1*), aminoglycoside resistance (*aac(69)-Ib-cr*, *aac(3)-II*, and *strB*), and quinolone resistance determinants (aminoglycoside acetyltransferase *aac(69)-Ib-cr* and *qnrB*). Based on sequence annotation, both pKPX-1 and pKPX-2 harbor genes related to conjugation functions. However, experimental findings indicate that pKPX-2 is indeed a conjugative plasmid, whereas pKPX-1 appears to lack the ability to conjugate.

With the spread of resistant plasmids, there has been an emergence of increasingly resistant strains of *K. pneumoniae*, which exhibit resistance to a broader range of antibiotics as these plasmids disseminate. Resistance genes such as *blaKPC*, *blaNDM*, *blaOXA*, *blaTME*, *blaSHV*, and *blaCTX-M* are located within the plasmids of *K. pneumoniae* and mediate the transmission of these genes. This phenomenon contributes to the widespread prevalence of bacterial resistance, undermines the effectiveness of antibiotics, and complicates the diagnosis and treatment of affected patients.

##### 2.4.1.2 IncN

**Figure 3:**
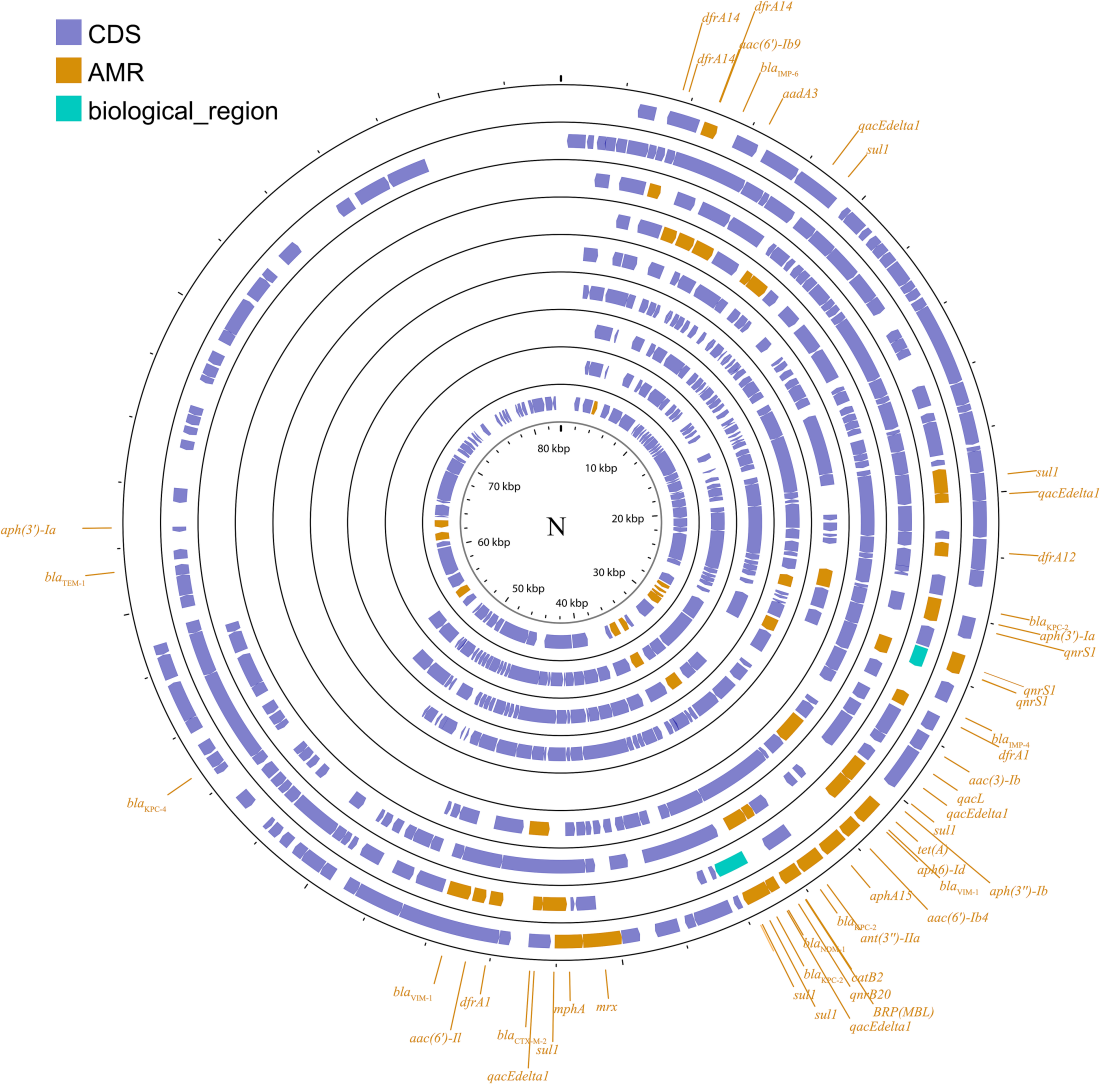
Plasmid profile of IncN. Including pBK31551, pFCF3SP, pFCF1305, pIMP-HZ1, pK186_KPC, pKPI-6, pNDM-BTR, pNL194, pOW16C2. Plasmids of the same plasmid group were placed in the same plasmid profile, and we marked their resistance genes and virulence genes in the plasmid profile. The sequence of plasmids in the plasmid profile from inside to outside is pBK31551, pFCF3SP, pFCF1305, pIMP-HZ1, pK186_KPC, pKPI-6, pNDM-BTR, pNL194, pOW16C2. CDS, coding DNA sequence; AMR, antimicrobial resistance.

IncN plasmids are typically broad host ranges and self-conjugative plasmids with a size ranging from 30 to 70 kb (García-Fernández et al., 2011). These plasmids have been associated with genes conferring resistance to ESBLs, sulfonamides, quinolones, aminoglycosides, tetracyclines and streptomycin. These groups have been described as three subgroups: IncN1, IncN2 and IncN3. The same collection method as above (Figure 3; Supplementary Figure S2) was used.

Imipenemase (IMP), an Ambler class B metallo-β-lactamase, is an important carbapenemase that confers resistance to almost all β-lactams. pIMP-4-BKP19 is a blaIMP-4-harboring IncN plasmid from a *K. pneumoniae* ST1873 strain discovered in China (Xu et al., 2020). The results of the conjugation experiment indicated that pIMP4-BKP19 does not possess conjugative abilities. However, it appears that the IS26-associated class 1 integron plays a role in disseminating the *blaIMP-4* gene to various plasmids. Another *blaIMP-4*-carrying IncN plasmid reported in China is pIMP-HZ1 (Lo et al., 2013). It is a conjugative plasmid equipped with qnrS1. Additionally, the West China Hospital discovered the presence of a self-transmissible IncN plasmid from *K. pneumoniae* strain WCHKP020034 harboring resistance genes: *blaNDM-1*, *dfrA14*, and *qnrS* (Liu et al., 2018). For *K. pneumoniae*, pNDM-BTR is also a *blaNDM-1*-encoding plasmid with a size of ~59.6 kb and belongs to IncN, which was found in Taiwan, China (Lai et al., 2018). IncN plasmid has been found in many countries. In Brazil, two *blaKPC-2*-carrying IncN plasmids (pFCF1305 and pFCF3SP) were isolated from *K. pneumoniae* strain ST442 (Pérez-Chaparro et al., 2014) and the *blaKPC*-containing conjugative IncN plasmids pBHKPC93_3 and pBHKPC104_3 from *K. pneumoniae* strains BHKPC93 and BHKPC104 were found to be transferable to *Escherichia coli* (Boralli et al., 2023). Similarly, IncN plasmids from *K. pneumoniae* have been reported to mediate the transfer of *blaKPC* among Enterobacteria in several Colombian hospitals (Rada et al., 2020). Additionally, the complete nucleotide sequencing of the self-transmissible plasmid pKPI-6, which encodes the *blaIMP-6* and *blaCTX-M-2* genes, was performed in Japan and revealed a classical IncN plasmid backbone (Kayama et al., 2015). The IncN plasmid pBK31551, originating from New Jersey, has a size of 84 kb and carries several resistance genes, including *blaKPC-4*, *blaTEM-1*, *qnrB2*, *aac(3)-Ib*, *aph (3*′*)-I*, *qacF*, *sul1*, and *dfrA14*. These genes confer resistance to various antibiotics such as β-lactams, quinolones, aminoglycosides, quaternary ammonium compounds, and co-trimoxazole (Chen et al., 2013). Furthermore, IncN plasmids have also been associated with *blaVIM* gene, as seen in a *blaVIM*-carrying IncN plasmid found at a university hospital in North Norway (Naseer et al., 2012). Similarly, pNL194, which encodes VIM-1 and was isolated from *K. pneumoniae* NL194, was found to be a self-transferable IncN plasmid (Miriagou et al., 2010). Overall, in different countries, IncN plasmids have been shown to mediate the dissemination of various antibiotic resistance genes in *K. pneumoniae*, enhancing its environmental adaptation and posing more challenges for clinical treatment.

##### 2.4.1.3 IncX

**Figure 4:**
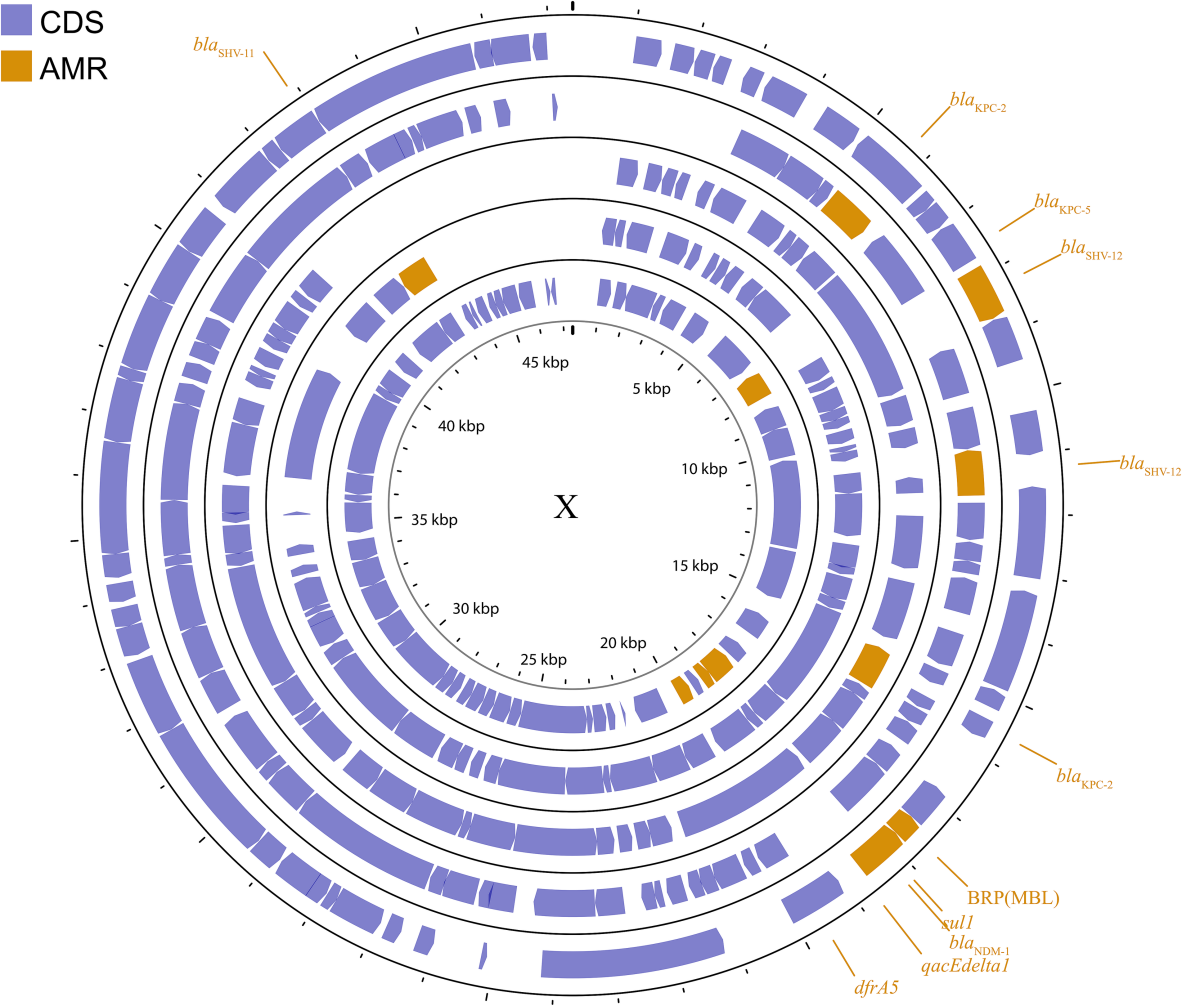
Plasmid profile of IncX. Including pBK31567, pIncX-SHV, pKPC-NY79, pKpS90, pNDM-HN380. Plasmids of the same plasmid group were placed in the same plasmid profile, and we marked their resistance genes and virulence genes in the plasmid profile. The sequence of plasmids in the plasmid profile from inside to outside is pBK31567, pIncX-SHV, pKPC-NY79, pKpS90, pNDM-HN380. CDS, coding DNA sequence; AMR, antimicrobial resistance.

The IncX plasmid is a narrow host range plasmid of the Enterobacteriaceae family that encodes type IV fimbriae, enabling self-conjugative transfer and providing auxiliary functions to host bacteria, such as resistance to antimicrobials and the ability to form biofilms (Johnson et al., 2012). The IncX group is usually associated with the transmission of *K. pneumoniae* carbapenemase genes, especially *blaKPC*, *blaNDM*, and *blaOXA-181* (Ho et al., 2013; Campos et al., 2015; Yang et al., 2015; Fortini et al., 2016; Kim et al., 2017). Several subgroups have been successively identified: IncX1, IncX2 (Jones et al., 1993), IncX3, IncX4 (Johnson et al., 2012), and IncX5 (Chen et al., 2013), among others. IncX3 is the major subgroup carrying *blaKPC* and *blaNDM* (Guo et al., 2022). The IncX plasmid pKpS90 is a transferable plasmid that carries *blaKPC-2* and *blaSHV-12* in *K. pneumoniae*, and its complete nucleotide sequence has been reported (Kassis-Chikhani et al., 2013). The plasmid pBK31567, which belongs to a novel IncX subgroup (IncX5), is 47 kb in length and harbors *blaKPC-5*, *dfrA5*, *qacEΔ1*, and *sul1* (Chen et al., 2013). The IncX group has not been valued in previous studies, but genetic analysis of it has revealed several surprising traits that were directly recruited from the chromosome of *K. pneumoniae* (Burmølle et al., 2012). The plasmids described above are shown in Figure 4; Supplementary Figure S3.

##### 2.4.1.4 IncA/C

**Figure 5:**
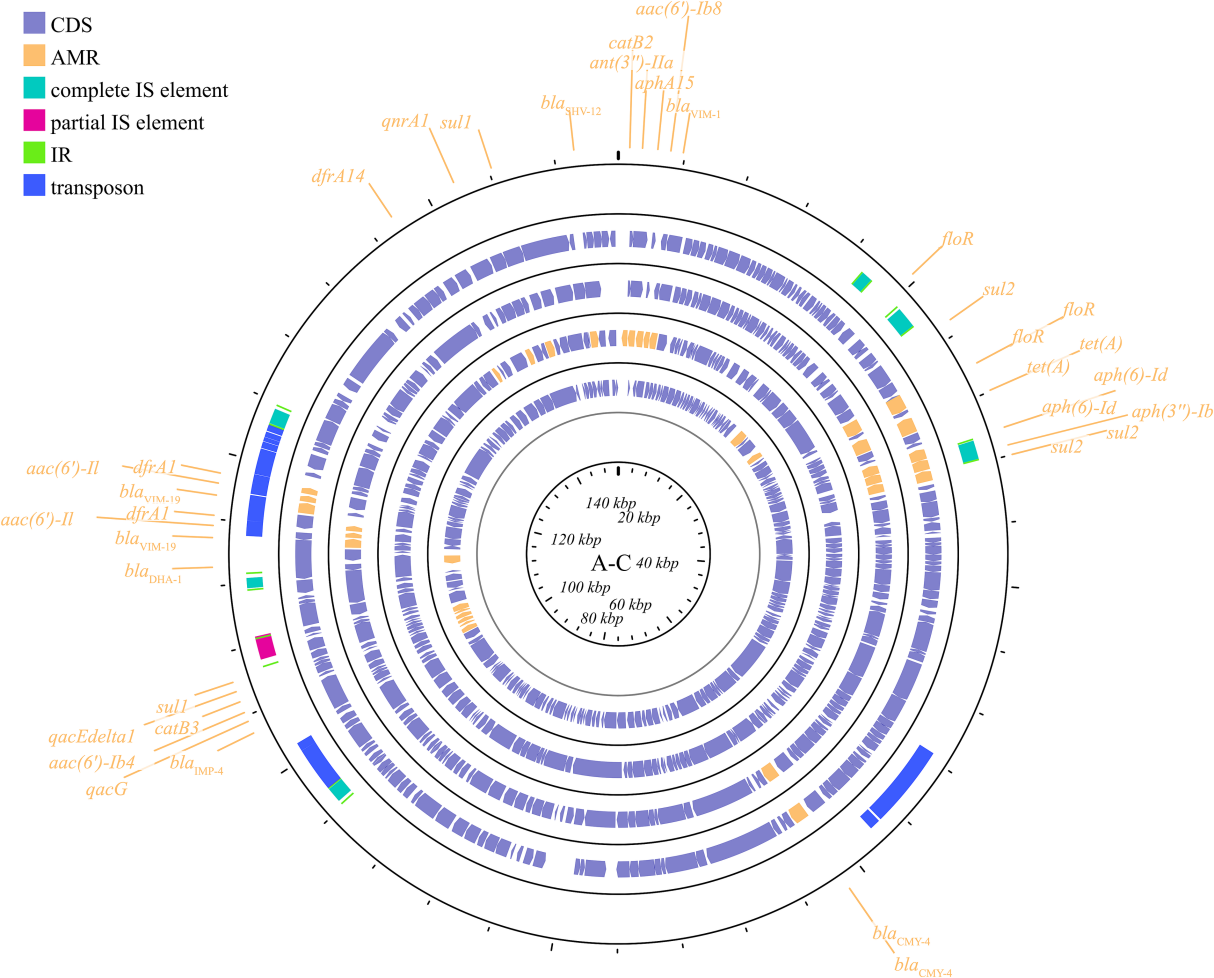
Plasmid profile of IncA-C. Including pIMP-PH114, pIncAC-KP4898, pKP-Gr642, pKP-Gr8143. Plasmids of the same plasmid group were placed in the same plasmid profile, and we marked their resistance genes and virulence genes in the plasmid profile. The sequence of plasmids in the plasmid profile from inside to outside is pIMP-PH114, pIncAC-KP4898, pKP-Gr642, pKP-Gr8143. CDS, coding DNA sequence; AMR, antimicrobial resistance.

IncA/C is a group of broad-host-range, low-copy-number, self-transferable plasmids in the size range of 40–230 kb, including two subgroups: A/C1 and A/C2 (Carraro et al., 2014). Although the coexistence of IncA and IncC has been demonstrated, they are categorized in the same incompatible plasmid group (Datta and Hedges, 1973; Hedges, 1974). IncA/C group plasmids have a significant role in the transmission of ARGs in *K. pneumoniae*. The IncA/C1 plasmid pIncAC_KP4898, carrying the extended spectrum β-lactamase *blaSHV-12* and carbapenem-hydrolyzing metallo-β-lactamase *blaVIM-1* genes, contributed to the transmission of ARGs in *K. pneumoniae* at the Naples Hospital (Esposito et al., 2017). Additionally, the discovery of pKP-Gr642 (162,787 bp) and pKP-Gr8143 (154,395 bp), containing *blaCMY-2* and *blaVIM-19*, respectively, belonging to the IncA/C2 subgroup in *K. pneumoniae* strain ST384 from Greece, has been reported (Papagiannitsis et al., 2016a). Likewise, the IncA/C2 subgroup plasmid carrying *blaIMP-4* gene, pIMP-PH114, was reported in the Philippines (Ho et al., 2014). In Taiwan, the IncA/C plasmid is the main plasmid type responsible for the transmission of ESBL genes in *K. pneumoniae*, along with the presence of *blaOXA-48* (Ma et al., 2015; Chang et al., 2017). Furthermore, the IncA/C plasmid containing *blaNDM-1* has been found in *K. pneumoniae* collected from outpatients in Thailand (Assawatheptawee et al., 2023). The conjugative plasmids of the IncA/C group pose a substantial threat due to their wide host range, the extensive spectrum of antimicrobial resistance they impart, their prevalence in enteric bacteria, and their highly efficient spread through conjugation (Carraro et al., 2014). These plasmids are visible in Figure 5 and Supplementary Figure S4.

##### 2.4.1.5 IncL/M

**Figure 6:**
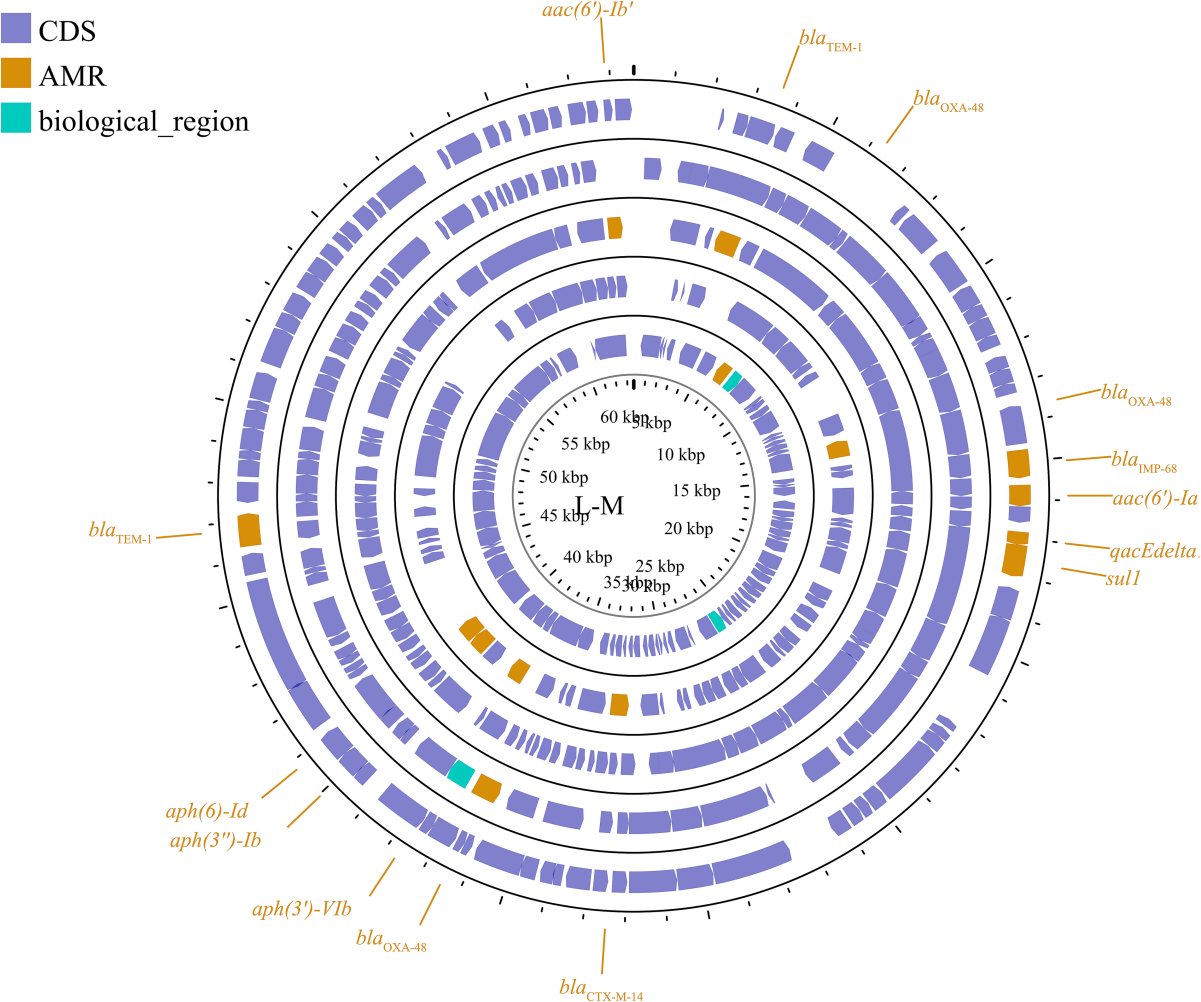
Plasmid profile of IncL/M. Including pE71T, pEGY22_CTX-M-14, pFOX-7a, pKPoxa-48N1, pTMTA63632. Plasmids of the same plasmid group were placed in the same plasmid profile, and we marked their resistance genes and virulence genes in the plasmid profile. The sequence of plasmids in the plasmid profile from inside to outside is pE71T, pEGY22_CTX-M-14, pFOX-7a, pKPoxa-48N1, pTMTA63632. CDS, coding DNA sequence; AMR, antimicrobial resistance.

Another important broad-host-range incompatibility type is the L/M group, which consists of low-copy-number and transferable plasmids. IncL/M plasmids play an important role in mediating the transfer of *blaOXA-48* in *K. pneumoniae*, as reported in Germany, China, France, Italy, Croatia, Spain, South America, the Arabian Peninsula, and Ireland (Berger et al., 2013; Liu et al., 2015; Guo L. et al., 2016; Loucif et al., 2016; Pérez-Vázquez et al., 2016; Gaibani et al., 2017; Al-Baloushi et al., 2018; Jelić et al., 2018; Schwanbeck et al., 2021; Salazar et al., 2022). In addition to carrying *blaOXA-48*, IncL/M plasmids have been reported to carry other ARGs in *K. pneumoniae*, such as *blaKPC-2* in a Chinese teaching hospital, *blaIMP-68* in Japan, *blaCTX-M-3* in Bulgaria, and *blaCTX-M-14* in Egypt (Markovska et al., 2014; Liu et al., 2015; Kubota et al., 2019; Edward et al., 2022). These plasmids are shown in Figure 6; Supplementary Figure S5. This plasmid group is relatively small compared to the IncF, but it plays a significant role in driving resistance in *K. pneumoniae*, limiting the range of available antibiotics.

##### 2.4.1.6 Other incompatible groups

**Figure 7:**
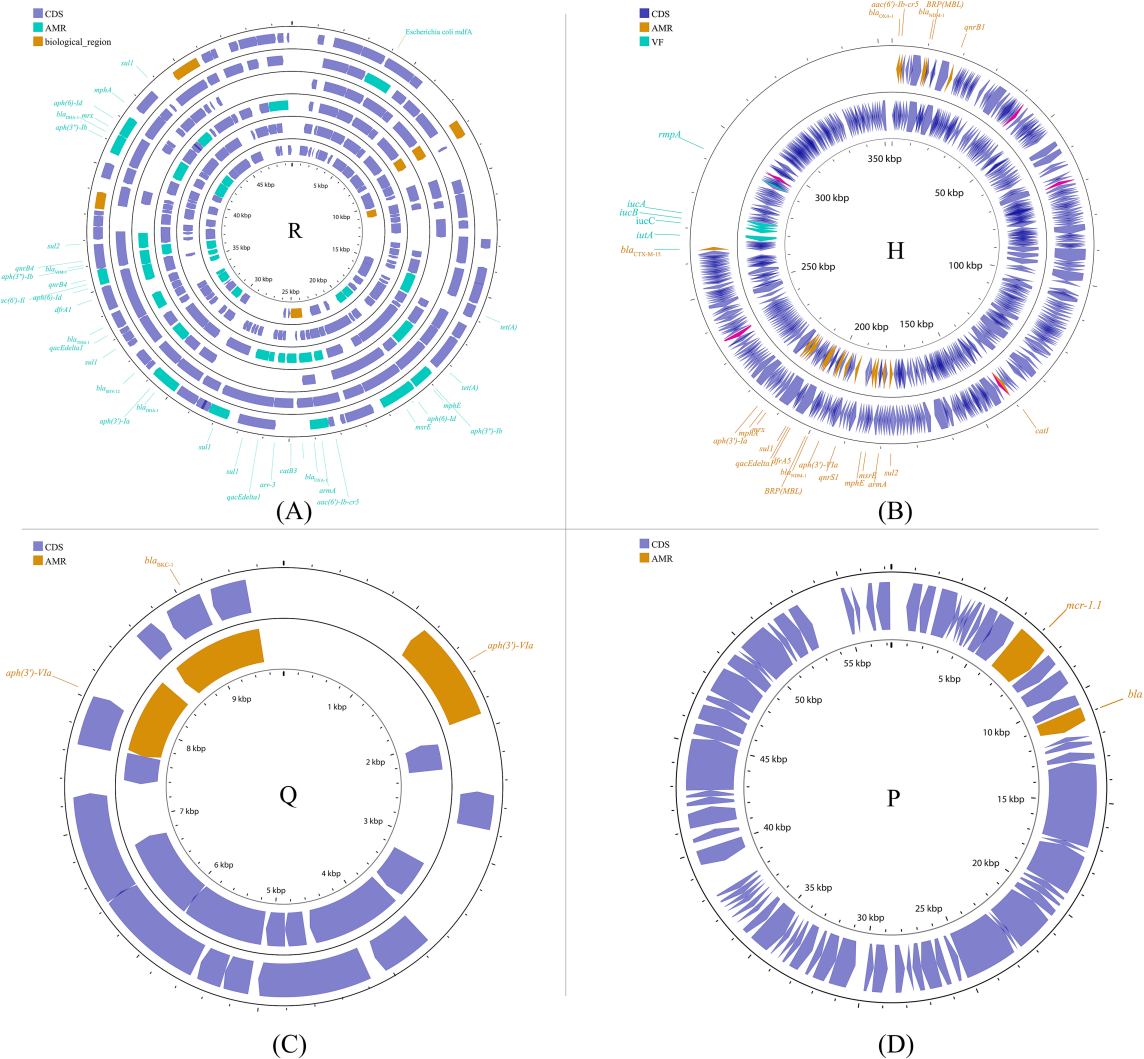
Plasmid profile of the other plasmid groups. Including IncR **(A)**: pKP1780, pKPC-LK30, pKPS30, pKPS77, pR50-74, pYDC676; IncH **(B)**: p51015_NDM-1, pNDM-MAR; IncQ **(C)**: p60136, pKPN535a; IncP **(D)**: pMCR_KP1511. Plasmids of the same plasmid group were placed in the same plasmid profile, and we marked their resistance genes and virulence genes in the plasmid profile. The sequence of plasmids in the plasmid profile from inside to outside is **(A)**: pKP1780, pKPC-LK30, pKPS30, pKPS77, pR50-74, pYDC676; **(B)**: p51015_NDM-1, pNDM-MAR; **(C)**: p60136, pKPN535a; **(D)**: pMCR_KP1511. CDS, coding DNA sequence; AMR, antimicrobial resistance; VF, virulence factor.

Other plasmid incompatibility groups also play a significant role in *K. pneumoniae* by mediating the spread of drug-resistant genes. Broad-host-range and mobilizable IncR plasmids are responsible for transferring multiple drug-resistant genes (Chen et al., 2006; Bielak et al., 2011; Compain et al., 2014; Guo Q. et al., 2016). Additionally, IncP, H, Y, and W plasmids have been reported to contribute to the transmission of drug-resistant genes (Almeida et al., 2012; Villa et al., 2012; Zhao et al., 2017; Ahmad et al., 2019) (Figure 7; Supplementary Figure S6).

#### 2.4.2 Virulence plasmid

Multiple virulence genes are present in the genome of *K. pneumoniae*, affecting bacterial adhesion, colonization, invasion, and growth on the surfaces of host organs or tissues. These genes establish mechanisms for immune evasion, allowing the bacteria to avoid destruction by the host’s immune system. Currently, the most extensively studied virulence factors include capsules, lipopolysaccharides, fimbriae, and iron uptake systems. All known capsule variants of *K. pneumoniae* are synthesized by the Wzx/Wzy polysaccharide polymerization machinery, which is encoded by a single capsule polysaccharide (CPS) biosynthesis locus (Pan et al., 2015). The regulator of mucoid phenotype A (*rmpA*), which encodes regulatory proteins for CPS synthesis, was first identified in *K. pneumoniae* as a determinant controlling CPS biosynthesis (Nassif and Sansonetti, 1986). The capsule polysaccharides of *K. pneumoniae* resist phagocytosis by obstructing phagocytic action and lack a recognition site (the 2,3-mannose structure) for macrophage surface lectins (Huang et al., 2022). Lipopolysaccharide is an endotoxin composed of lipid A, O-antigen, and an oligosaccharide core, encoded by the *lpx, wbb*, and *waa* gene clusters, respectively (Merino et al., 2000; Frirdich and Whitfield, 2005). The lipopolysaccharide O side chain of *K. pneumoniae* prevents complement C1q or C3b from binding to the bacterial cell membrane, thereby protecting the bacteria from complement-mediated membrane damage and cell death (Merino et al., 1992; Albertí et al., 1996; Zhang et al., 2017). In *K. pneumoniae*, type 1 and 3 fimbriae, encoded by the *fim* and *mrk* genes respectively, are the major adhesive structures that have been characterized as pathogenicity factors (Di Martino et al., 1996; Tarkkanen et al., 1998; Struve et al., 2008). Several siderophores are expressed in *K. pneumoniae*, including enterobactin (encoded by the *entABCDEF* genome), yersiniabactin (*irp* gene cluster), salmochelin (*iro* gene cluster), and aerobactin (*iucABCD* gene cluster) (Bach et al., 2000; Hsieh et al., 2008; Müller et al., 2009). They promote bacterial metabolism and are prerequisites for bacterial infection of the host.

Studies have shown that plasmids can mediate the dissemination of virulence genes, resulting in the emergence of HVKP, or even hypervirulent and drug-resistant *K. pneumoniae* (Jin et al., 2021). Generally, these virulence plasmids carry many virulence-encoding genes, including capsular polysaccharide (CPS) regulator genes (*rmpA* and *rmpA2*) and several siderophore gene clusters (iucABCD-iutA, iroBCDN, ybtAEPQTUX, and entABCDEFS clusters) (Struve et al., 2015). However, the classical virulence plasmids pLVPK and pK2044 are non-conjugative due to the absence of the tra gene, which is responsible for the conjugation transfer function (Yang X. et al., 2021). The specific mechanism by which pLVPK-like virulence plasmids are delivered remains unclear. Nevertheless, some studies have suggested that virulence plasmids can be transferred with the assistance of helper plasmids that contain all the necessary genes for self-transmission via conjugation (Yang et al., 2022). The transfer mechanisms include transfer alone, co-transfer with a conjugative plasmid, and hybrid plasmid formation resulting from two rounds of single-strand exchanges at specific 28-bp fusion sites or homologous recombination (Xu et al., 2021). This process is analogous to the mobilization process described earlier. Furthermore, the integration of a virulence-associated gene fragment from a virulence plasmid into a conjugative plasmid results in the formation of a new conjugative plasmid that may carry both resistance and virulence genes. This new plasmid plays a crucial role in the development of hypervirulent and drug-resistant *K. pneumoniae*.

The best-known virulence plasmids are the 219 kb plasmid pLVPK from *K. pneumoniae* CG43 (Chen et al., 2004) and the 224 kb plasmid pK2044 (Wu et al., 2009) from *K. pneumoniae* NTUH-K2044, with 96% sequence coverage and 99.39% homology, respectively (Hu, 2021). Virulence-associated genes identified in pLVPK include the CPS synthesis regulator gene *rmpA* and its homolog *rmpA2*, as well as multiple iron-acquisition system genes, including *iucABCD*, *iutA*, *iroBCDN*, *fepBC*, and *fecIRA*. Additionally, several gene clusters homologous to copper, silver, lead, and tellurite resistance genes found in other bacteria were also identified. Furthermore, the presence of high concentrations of heavy metals within cells can lead to the formation of nonspecific complex compounds, resulting in toxic effects.

**Figure 8:**
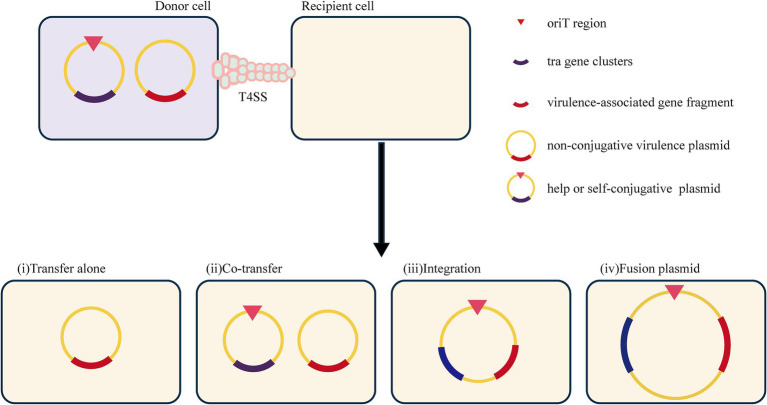
Potential mechanisms for the transfer of virulence genes in non-conjugative.

Although the classical virulence plasmid itself is incapable of mediating the dissemination of virulence-related genes via conjugation, some reports show that fragments of the virulence plasmid can be integrated into other plasmids to generate a conjugative virulence plasmid that can be transferred. For example, the virulence plasmid p15WZ-82_Vir is a conjugative plasmid formed by the integration of a fragment of the pLVPK virulence plasmid into a conjugative IncFIB-type plasmid (Yang et al., 2019). It can be transferred in *K. pneumoniae* and is even able to be horizontally transferred into CRKP, leading to the emergence of hypervirulent drug-resistant strains. In addition, the fusion plasmid (p17ZR-91-Vir-KPC) is a conjugative plasmid encoding virulence and resistance formed by a non-conjugative pLVPK-like virulence plasmid and a conjugation-resistant plasmid, allowing the evolution of an ordinary strain into an HVKP (Xie et al., 2021). However, recombination between gene fragments and insertion elements serves as a mechanism for the formation of hybrid plasmids (Han et al., 2022). (Potential mechanisms can be found in Figure 8). The high expression of the adhesion-associated factor *rmpA* probably restricts the transfer of the virulence plasmid (Xu et al., 2021). Additionally, the virulence plasmid can be transferred with the support of outer membrane vesicles (Wang et al., 2022).

### 2.5 ICE and IME

Integrative and conjugative elements (ICEs) are prevalent mobile units carrying modules responsible for excision, maintenance, conjugative transfer, and integration into the genome of a new host (Johnson and Grossman, 2015). ICEs are plasmid-like MGEs that intercept chromosomal genes from the host, which are transferred to recipient cells via conjugation, and subsequently integrate into the recipient chromosome (Thomas and Nielsen, 2005). A key distinction between ICEs and plasmids is that ICEs typically do not replicate independently; rather, they integrate into the chromosome and replicate in conjunction with it. ICE gene clusters are classified into four classical modules based on their functions: integration and excision (int-xis), mating-pair formation (mpf), mobilization and processing (mob), and regulatory (reg). Additionally, ICEs contain hot spots (HS) and variable regions (VR) that may include exogenous genes. The processes of ICE segregation and integration are associated with the genes for dissociase (xis) and integrase (int), respectively (Hirano et al., 2011). The widespread presence of the yersiniabactin gene cluster and its associated integrative conjugative elements (ICEKp) has been documented among clinical isolates of *K. pneumoniae* (Lam et al., 2018; Octavia et al., 2019; Jati et al., 2023). ICEKp1, an integrative and conjugative element identified in *K. pneumoniae*, plays a significant role in the transmission of the high-pathogenicity island (HPI) and contributes to the genomic heterogeneity associated with *K. pneumoniae* pyogenic liver abscess (PLA) infections (Lin et al., 2008). ICEKp1 can be divided into three distinct regions: one region resembles the high-pathogenicity island of Yersinia species and encodes the siderophore yersiniabactin; the second region resembles a portion of the large virulence plasmid pLVPK and encodes the glycosidophile salmochelin (iroBCDN) and the capsular polysaccharide regulator RmpA; the third region encodes the type IV secretion system (T4SS) (Shen et al., 2019). Due to their critical role in the transfer of carbapenemase genes (Botelho et al., 2020), ICEs pose a significant threat to human health by potentially facilitating the emergence of hypervirulent, drug-resistant *K. pneumoniae*.

Integrative and mobilizable elements (IMEs) exhibit structural similarities to integrative conjugative elements (ICEs), paralleling the relationship between conjugative plasmids and mobilizable plasmids. Both IMEs and ICEs are classified as mobile genetic elements (MGEs) that possess their own excision and integration modules. IMEs can utilize the mating pair formation (Mpf) machinery of conjugative elements, whether plasmids or ICEs, to facilitate their transfer between bacterial cells (Guédon et al., 2017). Importantly, the IME should not be regarded as a disabled component of the ICE; rather, it functions as a hitchhiking passenger.

## 3 Mechanisms of horizontal gene transfer

**Figure 9:**
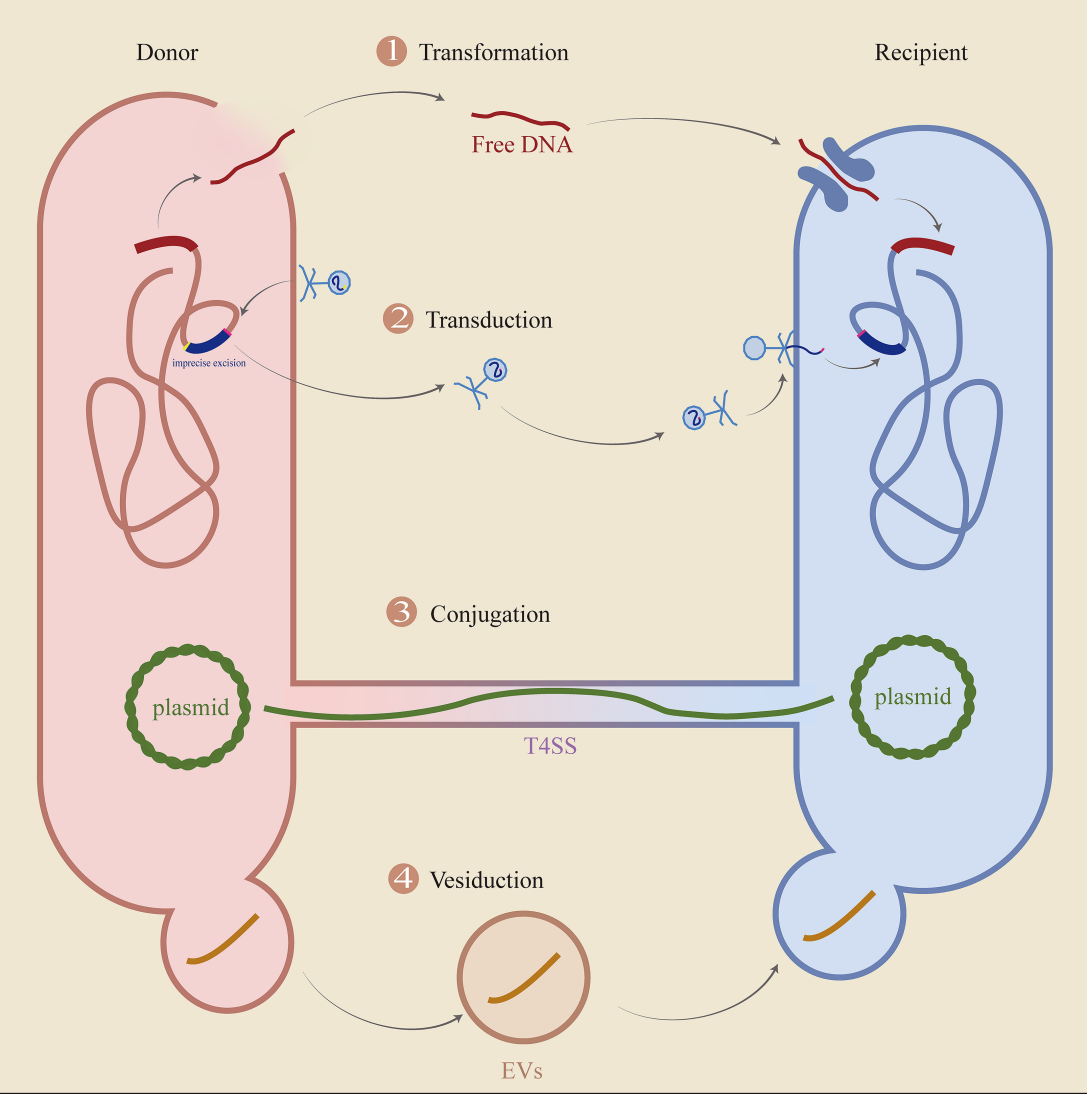
The key processes of HGT. The diagram delineates the fundamental processes involved in horizontal gene transfer (HGT), encompassing transformation, transduction, conjugation, and vesiduction. (1) Transformation: Cells are lysed, allowing DNA to be released and captured by recipient cells. (2) Transduction: The imprecise excision of the original bacteriophage takes genes from the donor cell, discards some of its genes, and then enters the recipient. (3) Conjugation: Transfer of plasmids or ICE via T4SS. (4) Vesiduction: Gene transfer mediated by vesicles, which is more conducive to protecting genetic material.

HGT is an important way for the expansion of drug resistance, and its main process is shown in Figure 9.

### 3.1 Transformation

Natural bacterial transformation refers to the process by which a recipient bacterium directly assimilates a specific DNA fragment containing genes from a donor bacterium present in the environment. The *blaIMP-68* gene, identified in *K. pneumoniae* strain TA6363 isolated in Tokyo, was inserted into the pHSG398 vector plasmid and subsequently used to transform *E. coli* DH5α cells. The minimum inhibitory concentration (MIC) of carbapenems was observed to increase in the recipient strain (Kubota et al., 2019). This finding suggests that when *K. pneumoniae* undergoes fragmentation, plasmids harboring resistance genes can escape and be transformed under specific conditions, facilitating the transfer of resistance genes.

This process can be delineated into four distinct sequential stages: competence induction, DNA uptake, translocation of DNA across the inner membrane (IM) and outer membrane (OM), and the integration or recombination of the incoming DNA into the genome or plasmid (Lorenz and Wackernagel, 1994; Averhoff et al., 2021). Research has been conducted to construct a comprehensive transformation mechanism by analyzing specific transformation processes in various bacterial species (Johnston et al., 2014). In a study by Averhoff et al. (2021), preliminary transformation models were developed for the systems of *Acinetobacter* and *Thermus thermophilus*. The transformation process can be summarized as follows: a bacterial pseudopilus penetrates a designated channel in the outer membrane and carries a DNA receptor at its tip, which enables recognition and binding of DNA from the external environment. Subsequently, the pseudopilus translocates the DNA through the secretin channel into the periplasm, where it is transported to the inner membrane by binding to a periplasmic protein. At this juncture, the DNA associates with an inner membrane protein that functions as a channel protein, facilitating the transfer of DNA into the cytoplasm. It is noteworthy that there are variations in the functional proteins involved in transformation systems across different species. In the extensively studied *Acinetobacter*, the functional proteins implicated in the transformation process include the fimbrillin proteins ComP, ComB, ComE, ComF, PilV, PilX, and FimT. The pili tip proteins are represented by ComC, while the secretin channel proteins consist of the multimeric secretin subunit known as PilQ. Furthermore, *Acinetobacter* employs the periplasmic binding protein ComEA and the endosomal protein ComA.

### 3.2 Transduction

Transduction is a biological process whereby a bacteriophage transfers genetic material from a host bacterium to another bacterium during the infection cycle. *K. pneumoniae* exhibits a high degree of lysogenicity and harbors prophages, which are likely associated with phage transduction (Wang et al., 2019; Kang et al., 2023). This genetic transfer involves the integration of the acquired genetic material into the genome of the recipient bacterium. The incorporation of the phage genome into the bacterial chromosome is referred to as lysogeny. Prior research has elucidated the mechanisms by which bacteriophages facilitate HGT (Borodovich et al., 2022). This overview aims to summarize key aspects of phage propagation, which are essential for understanding the transduction process. During replication, the phage genome undergoes replication and translation to produce structural and functional proteins, leading to the formation of concatemers. These concatemers are subsequently introduced into an empty viral capsid, where the terminase complex excises a copy for packaging. The filled capsid then assembles with the tail to form a progeny phage, while the terminase complex binds to the next empty capsid to continue the packaging process (Rao and Feiss, 2015). Transduction can be categorized into three distinct types based on the mechanisms involved: (i) specialized transduction, which occurs in temperate phages when the prophage integrated into the bacterial chromosome undergoes imprecise excision, resulting in the replication and packaging of adjacent host chromosomal genes into the phage. This process is restricted to genes located near the prophage and may lead to the loss of phage genes; (ii) generalized transduction, primarily observed in pac-type phages, involves the terminase complex recognizing a homologous region at the pac site within the host genome, excising a gene fragment of phage genome size, and packaging it into the phage; and (iii) lateral transduction, where the phage genome is replicated within the host chromosome without excision. In this case, the terminase complex acts on the pac site located in the middle of the phage genome, packaging the replicated DNA into daughter phages that contain both host and phage genes, resulting in multiple phages that incorporate segments of the host genome.

### 3.3 Conjugation

Conjugation represents a significant mechanism of HGT that necessitates direct contact between bacterial cells. As previously noted, in *K. pneumoniae*, MGEs such as conjugative plasmids and conjugation elements (ICEs) can be exchanged between bacterial cells through a cell-to-cell conjugation system (Navon-Venezia et al., 2017). This process is facilitated by various modules encoded by the plasmid, which include genes responsible for the type IV secretion system (T4SS), the T4SS coupling protein (T4CP), relaxosome accessory factors (RAF), and relaxase genes. Further details regarding the F-plasmid conjugation process and the roles of the associated molecules have been comprehensively documented by Virolle et al. (2020).

The initial phase of conjugation involves the expression of the tra gene, which is situated within the plasmid transfer region. This gene is responsible for the synthesis of all proteins associated with the conjugative pilus and the T4SS required for the establishment of the mating pair. Additionally, the tra gene produces components of relaxase that facilitate the processing of the plasmid before its transfer. In the subsequent phase, conjugative pilus is generated, which senses the extracellular environment and identifies recipient cells, thereby facilitating their aggregation with donor cells to form the mating pair (Curtiss, 1969; Folkhard et al., 1979; Willetts and Skurray, 1980; Clarke et al., 2008). Notably, it has been observed that the pilus may also serve as a conduit for the direct transfer of single-stranded DNA (Brinton, 1965). Following the establishment of the mating pair, the relaxosome—a complex comprising relaxases and auxiliary proteins—initiates the processing of the plasmid, which includes relaxosome cleavage and the isolation of T-DNA (Lanka and Wilkins, 1995; Dostál and Schildbach, 2010). The relaxosome binds to and cleaves a specific site known as the nik site located in the origin region of the plasmid, resulting in the relaxation of the double-stranded DNA. The processing of the plasmid entails the cleavage by the relaxosome and the subsequent isolation of the T-DNA (Cohen et al., 1968; Willetts and Skurray, 1980). The T-DNA subsequently forms a covalent bond with the relaxase, which, in conjunction with the Type IV coupling protein (T4CP) anchored to the cell membrane, facilitates its recruitment to the conjugative pore (Curtiss, 1969; de la Cruz et al., 2010). Thereafter, the T-DNA and relaxase are transferred to the recipient cell through the conjugation pore, with the leading sequence of the plasmid being the first to be transferred to the recipient (Frost et al., 1994). This concludes the description of plasmid transfer.

In the donor cell, the single-stranded cyclic plasmid is restored to its original structure through the process of rolling circle replication (RCR) (Waters and Guiney, 1993). In contrast, upon transfer to the recipient cell, the T-DNA initially forms a cyclized single-stranded DNA via the ligation of both ends of the oriT, a process facilitated by relaxases (Draper et al., 2005; Chandler et al., 2013). Subsequently, this cyclized single-stranded DNA is converted into double-stranded DNA through the coordinated action of the host’s DNA and RNA polymerases (Baharoglu and Mazel, 2014). In response to the incoming T-DNA, the host activates various defense mechanisms, perceiving it as exogenous DNA. These defense mechanisms encompass restriction modifications, nucleic acid exonucleases, recombinant systems, and adaptive immunity (Casjens, 2003). Importantly, the host’s single-stranded binding protein (SSB) plays a crucial role in protecting the T-DNA from nuclease degradation by enveloping it (Virolle et al., 2020). Additionally, the plasmid may encode PsiB proteins that inhibit SOS induction, thereby providing further protection for the plasmid (Bailone et al., 1988).

### 3.4 Vesiduction

Extracellular vesicles (EVs), which are generated through cellular metabolic activities, are spherical nanoparticles that encapsulate nucleic acids, proteins, lipids, and various other biomolecules (Kalluri and LeBleu, 2020). These vesicles play a crucial role in evading the immune response, transmitting virulence factors, facilitating intercellular communication, mediating HGT, facilitating nutrient and electron transfer, and promoting biofilm formation. Bacteria secrete a diverse array of extracellular vesicles, including outer membrane vesicles (OMVs), outer-inner membrane vesicles (OIMVs), and explosive outer membrane vesicles (EOMVs) (Toyofuku et al., 2019).

OMVs are nanoscale proteoliposomes characterized by a single membrane and are secreted by bacteria. These vesicles predominantly consist of components derived from the bacterial outer membrane and the periplasmic space (Amano et al., 2010; Ellis and Kuehn, 2010; Toyofuku et al., 2023). OMVs facilitate the differential intracellular release of various toxins and virulence factors, including adhesins, invasins, outer membrane proteins, lipopolysaccharides (LPS), flagellin, and proteases. Additionally, they may encapsulate cytoplasmic proteins and DNA (Kulp and Kuehn, 2010). Recently, a novel class of double-membrane bilayer EVs, known as outer-inner membrane vesicles (OIMVs), has been identified in Gram-negative bacteria (Pérez-Cruz et al., 2015). OIMVs not only contain the traditional components found in OMVs but also incorporate cytoplasmic elements, including DNA and plasmids. Consequently, OIMVs have been proposed as a significant type of EV involved in DNA (Toyofuku et al., 2019). The mechanisms underlying the production of EVs and the packaging of their contents remain inadequately understood; however, several hypotheses have been proposed regarding the presence of genetic material within these vesicles (Pérez-Cruz et al., 2013). The first hypothesis suggests that extracellular free DNA is internalized into the vesicles via a mechanism similar to bacterial transformation (Renelli et al., 2004). The second hypothesis posits that DNA is transported across the inner membrane and cell wall into the extraplasmic space, where it is subsequently encapsulated within the OMV (Kadurugamuwa and Beveridge, 1995). The third hypothesis, which has gained widespread acceptance, proposes that the formation of OIMVs involves the rolling of DNA from the cytoplasm of the bacterial cell (Pérez-Cruz et al., 2015). Thus, EVs, encompassing both OMVs and OIMVs, have the potential to facilitate horizontal gene transfer.

EVs have emerged as a novel mechanism of HGT in various bacterial species, functioning as carriers for the transfer of genetic material (Dorward et al., 1989; Yaron et al., 2000; Rumbo et al., 2011). These vesicles can adhere to the outer membrane of recipient cells, thereby facilitating the transmission of genetic elements between different bacterial species, which may result in the acquisition of new pathogenic traits or drug resistance. The uptake pathways for extracellular vesicles into host cells may involve several mechanisms, including macropinocytosis, clathrin-mediated endocytosis, caveolin-mediated endocytosis, or non-caveolin, non-clathrin-mediated endocytosis (O’Donoghue and Krachler, 2016). Research on *K. pneumoniae* has demonstrated that outer membrane vesicles (OMVs) play a critical role in mediating the transfer of drug-resistant or virulence plasmids both within and between bacterial species. Dell’Annunziata et al. (2021) was the first to report the transfer of plasmids containing resistance genes via OMVs derived from *K. pneumoniae*. Furthermore, research conducted by Wang et al. (2022) indicated that OMVs facilitate the transfer of the virulence plasmid phvK2115, which harbors various virulence genes, including *rmpAp*, *rmpA2p*, *iucA*, *iroB*, and *peg344*, within and among HVKP species. Additionally, it has been demonstrated that OMVs can disseminate two CRK3022 resistance plasmids, which contain genes such as *blaKPC-2* and *blaCTX-M-1*, among *K. pneumoniae* and *Escherichia coli* strains within the Enterobacteriaceae family (Tang et al., 2023). Notably, all of the aforementioned plasmids possess conjugation transfer regions, yet it remains unclear whether these regions play a corresponding role in the fusion of the outer vesicle with the recipient cell. OMVs isolated from *K. pneumoniae* NUHL30457 (K2, ST86) have also been shown to transfer both resistance and virulence genes, resulting in a phenotype characterized by increased drug resistance and virulence (Li P. et al., 2022). Moreover, the plasmid IncFIBpKPHS1, which carries *blaNDM-1* within OMVs, is also transmitted among *K. pneumoniae* strains (Tang et al., 2023). It is important to note that the aforementioned studies must exclude the possibility of free plasmids entering *K. pneumoniae* through transformation during the experimental procedures. Furthermore, the specific type of extracellular vesicles involved has not been delineated; it remains uncertain whether they are single-membrane OMVs or double-membrane outer-inner membrane vesicles (OIMVs). Therefore, future investigations should aim to differentiate between these vesicle types to enhance our understanding of the mechanisms by which genetic material enters outer membrane vesicles and to ascertain which type of outer membrane vesicles predominantly mediates gene transfer.

EVs can mediate the transmission of drug-resistance genes and virulence genes between *K. pneumoniae*, presenting additional challenges for clinical treatment. However, EVs offer several advantages over traditional plasmid-mediated gene transfer (Bonnington and Kuehn, 2014). Firstly, EVs do not require direct cell-to-cell contact for long-distance transport, significantly enhancing transfer efficiency. Secondly, they protect the molecular biological components contained within them from external environmental factors, thereby improving the stability of these components. For instance, EVs safeguard DNA from degradation by extracellular nucleases (Pérez-Cruz et al., 2015).

## 4 MGEs relationships

The nested structure of these MGEs is analogous to the Russian doll model and exhibits cross-cutting relationships (Figure 1). In this context, MGEs are categorized into intracellular and intercellular types. Intracellular MGEs encompass gene cassettes, integrons, and transposon elements, which exhibit an inclusion relationship; specifically, gene cassettes can be integrated into integrons, and integrons may also form part of the transposon structure (Di Pilato et al., 2015). Conversely, intercellular MGEs include prophages, ICE/IME, and plasmids. These elements facilitate the horizontal transfer of genetic material between cells through mechanisms such as transduction and conjugation, thereby expressing the associated traits. As previously mentioned, intracellular MGEs possess the ability to mobilize across the host genome, allowing for their presence within intercellular MGEs. Notably, integrons and transposable elements can be found within prophages, ICE/IME, and plasmids during their transfer. Furthermore, outer vesicles, which have recently been identified as vehicles for the dissemination of genetic material, may also encapsulate a variety of the aforementioned MGEs.

## 5 Clinical relevance of MGEs in *Klebsiella pneumoniae*

On May 17, 2024, the WHO published an updated list of drug-resistant bacteria that are most threatening to human health. Carbapenem-resistant *K. pneumoniae* was ranked first in the critical priority group. MGEs are the culprits for the emergence of carbapenem-resistant *K. pneumoniae*, as they help spread resistance genes and virulence factors widely among bacteria (Naim et al., 2018).

Although CRKP poses a major health threat worldwide, there are differences in drug resistance patterns and treatment options in different countries and regions. In China, the main drug resistance mechanism of CRKP is plasmid-mediated antibiotic resistance genes, and the main resistance gene is *blaKPC-2*. Therefore, most CRKP strains are susceptible to tigecycline, polymyxin B and ceftazidime-avibactam (Hu et al., 2024). In the United States, the main resistance mechanism is also plasmid-mediated resistance gene transfer, mainly associated with pColKP3-type and Inc-type plasmids (Cerón et al., 2023). Initially, polymyxin B and colistin were recommended as treatment options (Cerón et al., 2023); however, the nephrotoxic effects of polymyxin B and colistin have led to a shift in treatment options to the use of ceftazidime-avibactam in combination with aztreonam or cefdirox for infections with NDM enzyme-producing *K. pneumoniae*; ceftazidime-avibactam alone for urinary tract infections that produce OXA-48 enzymes; while meropenem-vaborbactam, ceftazidime-avibactam, and imipenem-cilastatin-relebactam are the preferred treatment options for infections with Enterobacteriaceae that produce KPC enzymes (Tamma et al., 2024). In Europe, the emergence of CRKP is mainly associated with the *blaKPC* gene, followed by *blaOXA-48* and *blaNDM-1* (Budia-Silva et al., 2024) and the spread of these resistance genes is associated with IncF type and ColRNAI plasmids (Loconsole et al., 2020). However, the resistance genes in some European countries also show different manifestations: Romania and Turkey mainly show *blaNDM-1*, Spain mainly shows *blaKPC* genes, Serbia mainly shows *blaOXA-48*, while Greece and Italy show a large number of *blaKPC* and other resistance genes, including *blaVIM, blaNDM* and *blaOXA-48*, Colistin is the last resort antibiotic for the treatment of Enterobacteriaceae infections (Loconsole et al., 2020; Afolayan et al., 2023). In Russia, the main resistance genes include *blaOXA-48*, followed by *blaNDM-1* and *blaKPC-3*, and colistin is the main treatment option (Shamina et al., 2020). In India, *blaOXA-48*-like genes are the most common, followed by *blaNDM-1* and *blaNDM-5* (Nagaraj et al., 2021). More detailed information on drug resistance patterns, current treatment options in different regions, and the transmission patterns of drug resistance genes and virulence genes. They are shown in Table 2.

**Table 2:** Summary of MGEs and their associated ARGs, virulence genes, regional distribution, and treatment options.

MGE	Name	Carbapenemase gene	Other resistance	virulence	Inc	Treatment options	Region	References
IS	ISKpn14	blaKPC-2	mgrB	—	—	Meropenem-vaborbactam, ceftazidime-avibactam, and imipenem-cilastatin-relebactam	China	Li et al. (2024)
	ISKpn25	blaKPC-3	mgrB	—	—		Medellín, Colombia	Cienfuegos-Gallet et al. (2017)
	ISKpn26	blaKPC-2	—	wcaJ	—		China	Yang Y. et al. (2021)
	ISKpn74		mgrB	—	—		China	Pu et al. (2023b)
	IS26	blaNDM-5	—	—	—	Ceftazidime-avibactam in combination with aztreonam, or cefiderocol	China	Yang et al. (2022)
Tn	Tn6296	blaKPC-135	—	—	—	Ceftazidime-avibactam, meropenem-vaborbactam, imipenem-cilastatin-relebactam, ceftolozane-tazobactam, and cefiderocol	China	Shi et al. (2024)
	Tn4401a	blaKPC-2/-3/-23	—	—	—		Korea/Italy/Greece	Fortini et al. (2016), Jeong et al. (2018), and Galani et al. (2019)
	Tn4401b	blaKPC-3	—	—	—		Italy	Garbari et al. (2015)
	Tn4401c	blaKPC-2	—	—	—		Korea	Yoon et al. (2018)
	Tn6454	blaKPC-2	—	—	—		Bogota, Colombia	Abril et al. (2021)
	Tn1999.2	blaOXA-48	—	—	—	Ceftazidime-avibactam	China	Li W. et al. (2022)
	Tn2016	blaOXA-204	—	—	—		Tunisia	Mansour et al. (2017)
	Tn125	blaNDM-1	—	—	—	Ceftazidime-avibactam in combination with aztreonam, or cefiderocol	China	Li et al. (2020)
	Tn6404	blaIMP-4	—	—	—		China	Zhou et al. (2017)
	Tn1331	—		BanHI	—	—	Portland	Tolmasky and Crosa (1987)
	Tn2012	blaCTX-M-15	—	—	—	Ceftazidime-avibactam, meropenem-vaborbactam, imipenem-cilastatin-relebactam, ceftolozane-tazobactam, and cefiderocol	China	Tian et al. (2022)
Plasmid	pKpS90	blaKPC-2	blaSHV-12	—	X	Meropenem-vaborbactam, ceftazidime-avibactam, and imipenem-cilastatin-relebactam	French	Kassis-Chikhani et al. (2013)
	pKP048	blaKPC-2	qnrB4, armA	—	FIIK		China	Jiang et al. (2010)
	p0716-KPC	blaKPC-2	blaTME-1	—	FII		China	Feng et al. (2017)
	p12181KPC	blaKPC-2	—	—	FII			
	pFCF3SP	blaKPC-2	—	—	N		Brazil	Pérez-Chaparro et al. (2014)
	pGR-1780	blaKPC-2	—	—	F		Greece, Heraklion	Papagiannitsis et al. (2016a)
	pBHKPC93_3	blaKPC	—	—	N		Brazil	Boralli et al. (2023)
	pBHKPC93_4	blaKPC	—	—	N			
	pBK31567	blaKPC-5	sul1		X		New Jersey	Chen et al. (2013)
	pFCF1305	blaKPC-2	—	—	N		Brazi	Pérez-Chaparro et al. (2014)
	pBK31551	blaKPC-4	blaTEM-1 qnrB2		N		New Jersey	Chen et al. (2013)
	pNL194	blaVIM-1	aacA7	—	N	Ceftazidime-avibactam in combination with aztreonam, or cefiderocol	Athens, Greece	Miriagou et al. (2010)
	pKP-Gr642	blaVIM-19	blaCMY-2	—	A/C		Greece	Papagiannitsis et al. (2016b)
	pKP-Gr8143	blaVIM-19	blaCYM-2	—	A/C			
	pIncAC_KP4898	blaVIM-1	blaSHV-12	—	A/C		Naples, Italy	Esposito et al. (2017)
	pKPI-6	blaIMP-6	CTX-M-2	—	N		Japan	Kayama et al. (2015)
	pIMP-HZ1	blaIMP-4	qnrS1	—	N		China	Lo et al. (2013)
	pIMP-PH114	blaIMP-4	aacA4	—	A/C		Philippine	Ho et al. (2014)
	p0801-IMP	blaIMP-1	—	—			China	Jiang et al. (2017)
	pIMP-4-BKP19	blaIMP-4	—	—	N		China	Xu et al. (2020)
	pNDM-BTR	blaNDM-1	—	—	N		China	Zhao et al. (2017)
	pKpn35963cz	blaNDM-1	—	—	A/C		Czech	Paskova et al. (2018)
	pKPX-1	blaNDM-1	—		F		China	Huang et al. (2013)
	pNDM-1-IncFIB-KPN-Spain	blaNDM-1 blaOXA-1	blaCTX-M-15	—	FIB		Spain	—
	pOXA48a	blaOXA-48	—	—	L	Ceftazidime-avibactam	Ireland	Power et al. (2014)
	pKPoxa-48 N1	blaOXA-48	—	—	—		China	Power et al. (2014)
	pKPoxa-48 N2	blaOXA-48	—	—	—			
	pKPX-2	blaOXA-1	blaTEM-1 blaCTX-M-15	—	F		China	—
	pKP96	—	CTX-M-24	—	FII	Ceftazidime-avibactam, meropenem-vaborbactam, imipenem-cilastatin-relebactam, ceftolozane-tazobactam, and cefiderocol	China	Shen et al. (2008)
	p17ZR-91-Vir-KPC	blaKPC-2	blaSHV-2, oqxAB, mph(A), sul1, aadA2, dfrA12	rmpA, rmpA2, iucABCD, iutA, iroBCDN, ybt9; mrkABCDFHIJ,	H	Meropenem-vaborbactam, ceftazidime-avibactam, and imipenem-cilastatin-relebactam	China	Xie et al. (2021)
	pCRHV-C2244	blaKPC-2	blaCTX-M-65	iroBCDN, iucABCDiutA, rmpA2	—	Meropenem-vaborbactam, ceftazidime-avibactam, and imipenem-cilastatin-relebactam	China	Jin et al. (2021)
ICE	ICEKp1	—	—	vagC, vagD, iroBCDN, rmpA	—	—	Singapore	Octavia et al. (2019)
	ICEKp2,4,9, 11	—	—	ybt	—	—		
	ICEKp3	—	—	ybt, iuc, iro, rmpA, rmpA2	—	—		
	ICEKp10	—	—	ybt, clb, iuc, iro, rmpA, rmpA2	—	—		

The emergence of hypervirulent drug-resistant *K. pneumoniae* poses a significant public health threat, driven by three primary pathways: (i) the acquisition of virulence plasmids by drug-resistant strains, (ii) the transfer of drug-resistant plasmids to highly virulent strains, and (iii) the simultaneous acquisition of both drug-resistant and virulence plasmids by cKP strains. The rise of the “superbug” CR-hvKP is particularly concerning due to the limited therapeutic options available (Pu et al., 2023a). Potential treatment strategies for CR-hvKP infections include novel combinations of β-lactam antibiotics with β-lactamase inhibitors, as well as the combination of polymyxin B with minocycline or rifampicin, and the use of zidovudine in conjunction with rifampicin (Lei et al., 2024). However, prolonged use of these antibiotics may lead to emergence of new resistant strains, such as polymyxin-resistant variants (Chen X. et al., 2022). Therefore, there is an urgent need for research and development of novel antibiotics, alongside a focus on rational and standardized antibiotic stewardship.

Given the ongoing emergence of “superbug” strains and the escalating threat to antimicrobial defenses, it is imperative to prioritize the development and implementation of infection prevention and control policies for CR-hvKP, alongside robust antibiotic stewardship programs. Effective antimicrobial stewardship involves the optimal selection, dosage, and duration of an antimicrobial agent that yields the best clinical outcomes for the treatment or prevention of infections, while minimizing toxicity to the patient and reducing the potential for subsequent resistance (Dyar et al., 2017). In the absence of antibiotic pressure, it is plausible that we could effectively prevent the emergence of drug-resistant strains at their source. The implementation of infection prevention strategies is particularly critical in the context of drug-resistant strains (Okeah et al., 2021). The study implemented several strategies to manage patients colonized or infected with CRKP: (1) isolating of affected patients in individual rooms; (2) cohorting CRKP patients with specialized nursing staff, along with screening patients in proximity to newly identified CRKP carriers; (3) executing of weekly active surveillance for patients in the intensive care unit; and (4) selectively surveilling of patients admitted to the emergency department. To evaluate the impact of these interventions on the CRKP epidemic, interrupted regression analysis and change-point analysis were employed (Cohen et al., 2011).

## 6 Challenges and future directions

With the continuous deepening of research on mobile genetic elements (MGEs), their transmission mechanism has become clearer, but their research is also facing more and more challenges. With the frequent and intensified transmission and combination between MGEs, the diversity and structural complexity of MGEs make detection more and more complicated. For example, metagenomic assembly is considered to be the main bottleneck of MGE identification. The existing MGE database is not comprehensive and cannot cover all types of MGEs, resulting in many MGEs being unable to be accurately annotated. It is not yet fully clear whether the interactive transmission of mobile genetic elements in different hosts is affected by the host’s immune system and physiological state, as well as the long-term ecological and evolutionary impact on the host. The uncertainty of cross-species transmission increases the complexity of drug resistance transmission and the difficulty of prevention and control. In addition, some potential mechanisms of MGE transmission have not been fully elucidated, including the process of extracellular vesicles encapsulating genetic material and its interaction mechanism with target cells, which need to be further clarified. These issues pose major challenges to the evolutionary study of MGEs and the prevention and control monitoring of antibiotic resistance. Therefore, exploring unknown types of MGEs, revealing their structure and function; studying the interaction between MGEs and host genomes, revealing their impact on host adaptability and evolution; and evaluating the impact of MGEs spread in different ecosystems on global health and ecological balance will become the direction of our future research. Through technology optimization and interdisciplinary cooperation, the development of resistance transmission mechanisms and prevention and control strategies, MGEs research is expected to provide new ideas and methods for solving resistance problems and promoting the development of genome engineering technology.

## 7 Conclusion

The widespread spread of mobile genetic elements (MGEs) among *K. pneumoniae* has led to the emergence of multi-drug resistant *K. pneumoniae* (MDRKP) and carbapenem-resistant hypervirulent *K. pneumoniae* (CR-hvKP), which has seriously aggravated the global difficulties in the treatment and prevention of *Klebsiella pneumoniae*. Therefore, the monitoring and transmission mechanism of mobile genetic elements in *K. pneumoniae* should be strengthened, the barriers of different disciplines should be broken, the cooperation of researchers in different fields should be promoted, and exploration should be made in all aspects, such as detection technology, gene transmission, host influence, and MGEs evolution, to provide a new scheme for solving the problems of clinical diagnosis and treatment, prevention and control, and promoting the development of genome engineering technology of mobile genetic elements.
