# Supplementary material for: Mobile genetic elements encoding antibiotic resistance genes and virulence genes in Klebsiella pneumoniae: important pathways for the acquisition of virulence and resistance
Source: Front Microbiol. 2025 Feb 24;16:1529157. doi: 10.3389/fmicb.2025.1529157 (PMC11891212; doi:10.3389/fmicb.2025.1529157)
Supplement: Supplementary file 1 [file Image_1.pdf]

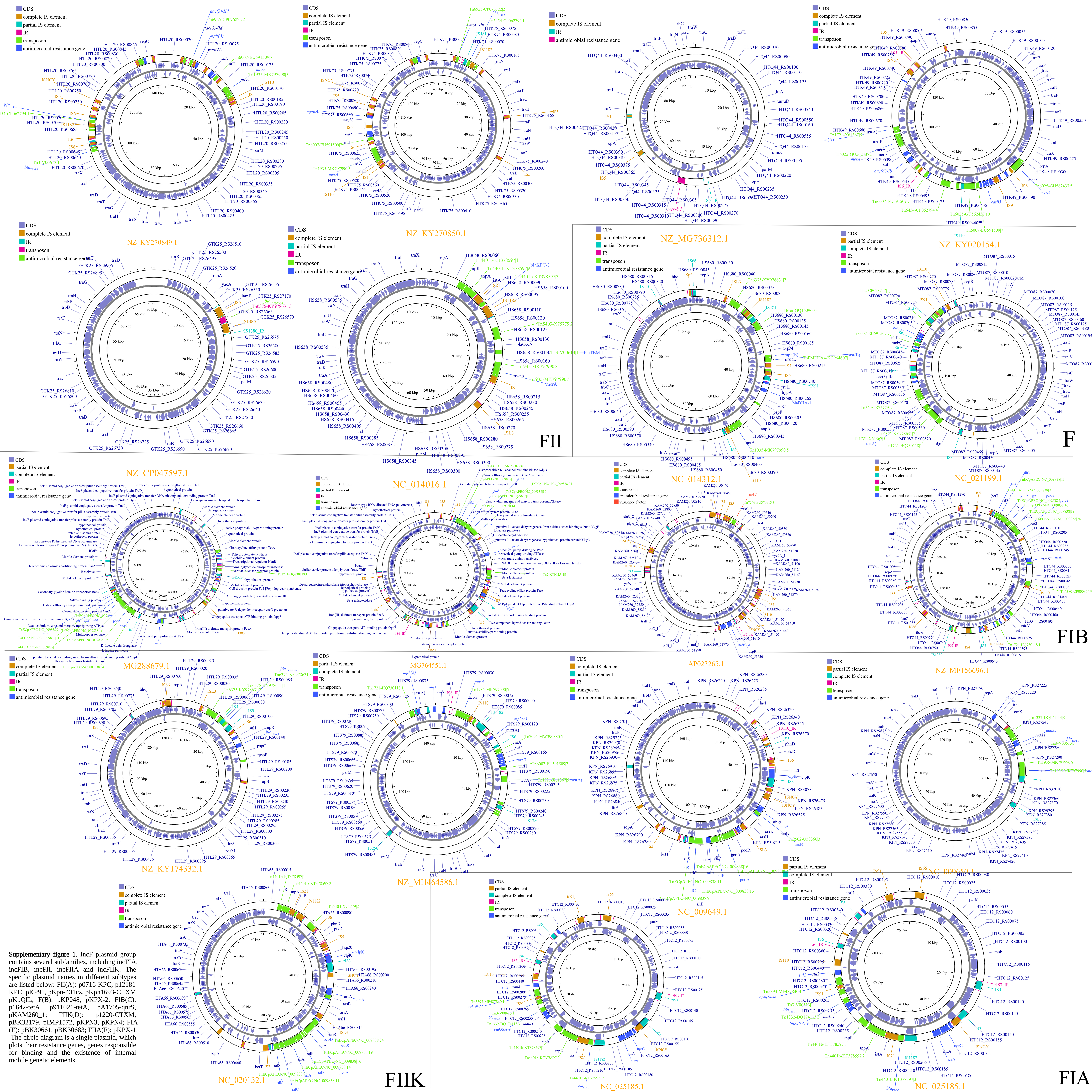

Supplementary figure 1. IncF plasmid group contains several subfamilies, including incFIA, incFIB, incFII, incFIIK and incFIIA. The specific plasmid names in different subtypes are listed below: FII(A): p0716-KPC, p12181-KPC, pKP91, pKpn-431cz, pKpn1693-CTXM, pKpQIL; F(B): pKP048, pKPX-2; FIB(C): p1642-tetA, p911021-tetA, pA1705-qnrS, pKAM260_1; FIIK(D): p1220-CTXM, pBK32179, pIMP1572, pKPN3, pKPN4; FIA (E): pBK30661, pBK30683; FIIA(F): pKPX-1. The circle diagram is a single plasmid, which plots their resistance genes, genes responsible for binding and the existence of internal mobile genetic elements.

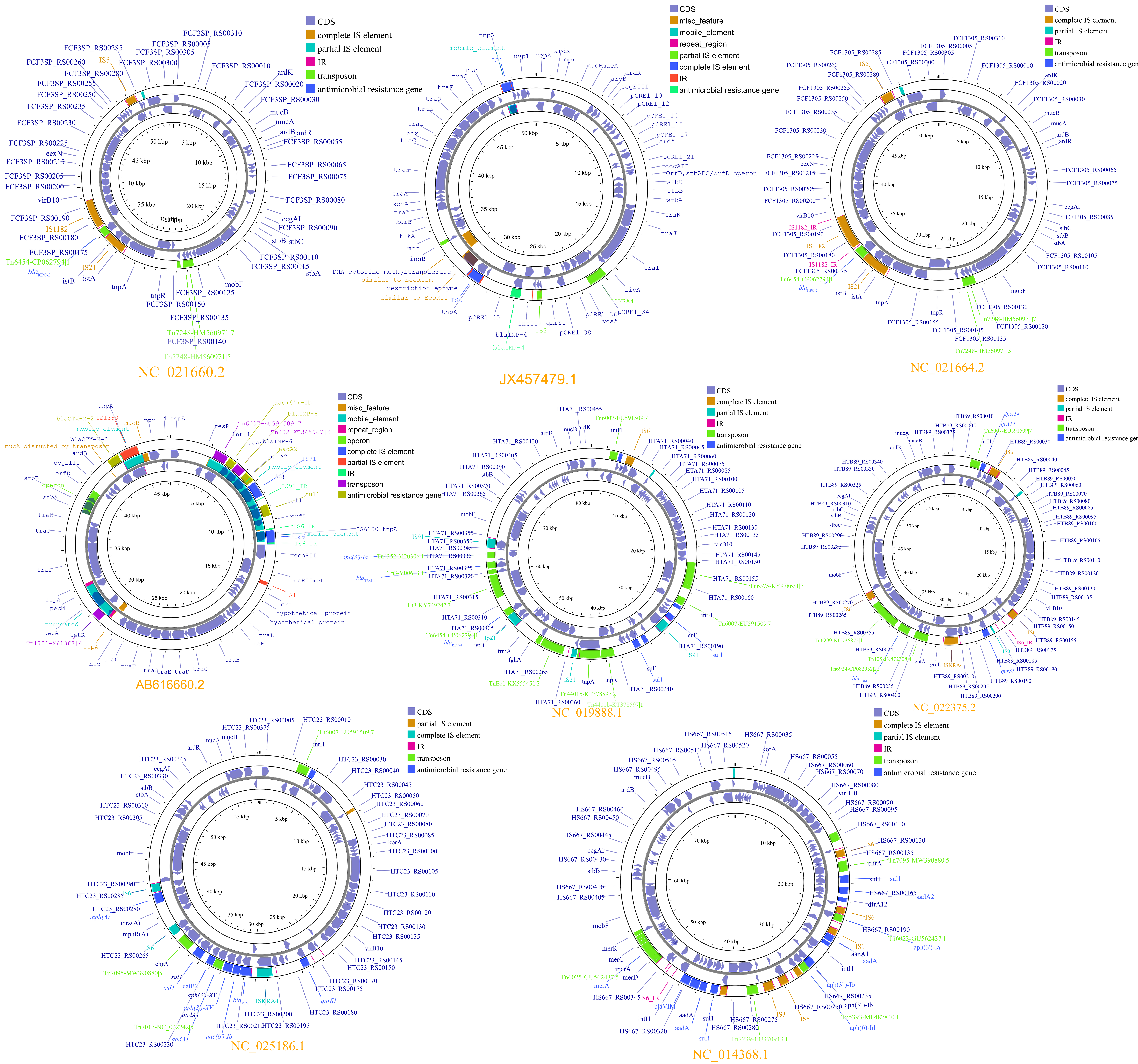

Supplementary figure 2. Plasmid map of IncN. Includs: pBK31551(B), pFCF3SP(C), pFCF1305(D), pIMP-HZ1(E), pK186_KPC, pKPI-6(F), pNDM-BTR(G), pNL194(H), pOW16C2(I). The circle diagram is a single plasmid, which plots their resistance genes, genes responsible for binding and the existence of internal mobile genetic elements.

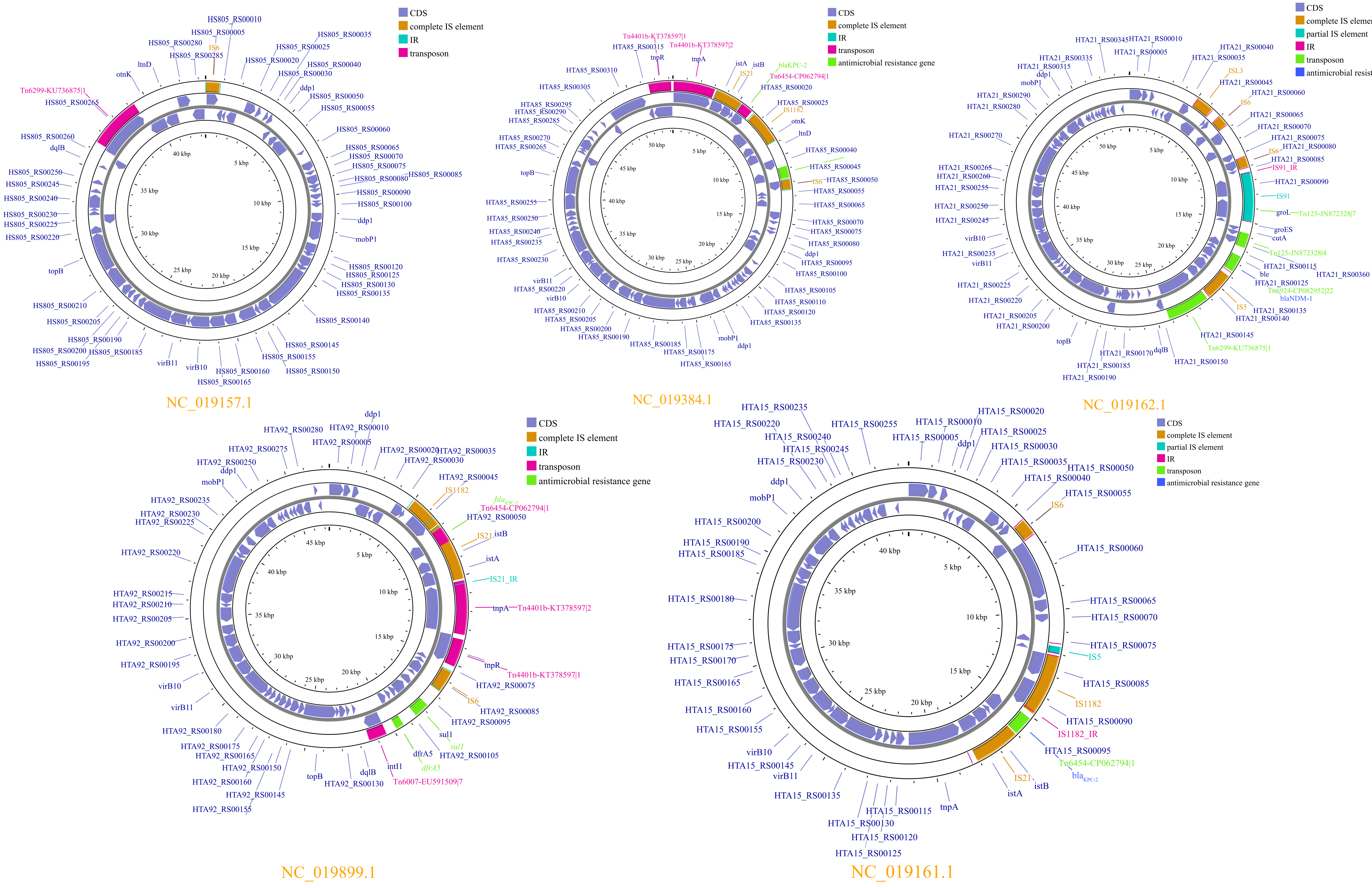

Supplementary figure 3. IncX: pBK31567(B), pIncX-SHV(C), pKPC-NY79(D), pKpS90(E), pNDM-HN380(F).The circle diagram is a single plasmid, which plots their resistance genes, genes responsible for binding and the existence of internal mobile genetic elements.

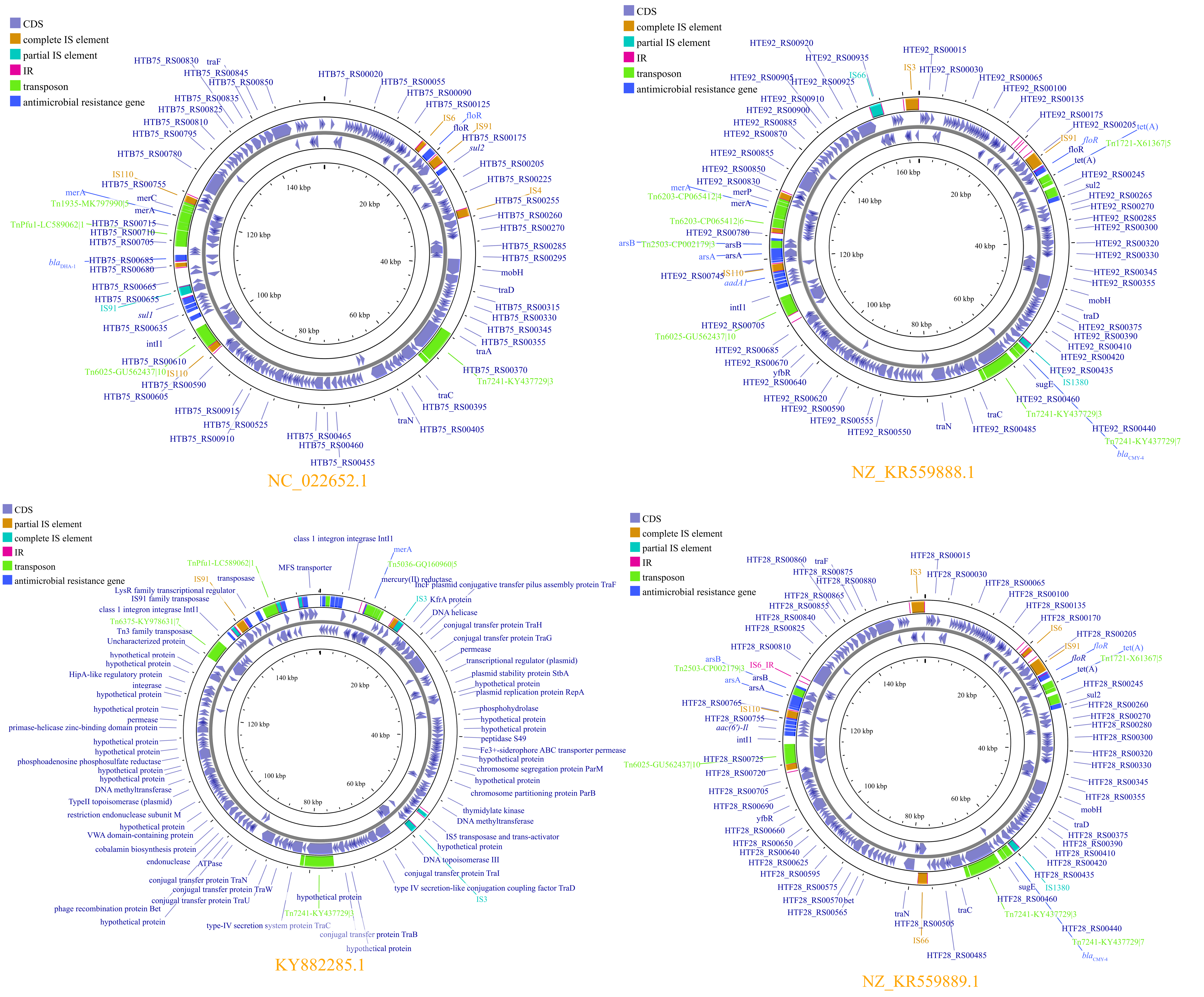

Supplementary figure 4. IncA-C: pIMP-PH114, pIncAC-KP4898, pKP-Gr642, pKP-Gr8143.The circle diagram is a single plasmid, which plots their resistance genes, genes responsible for binding and the existence of internal mobile genetic elements.

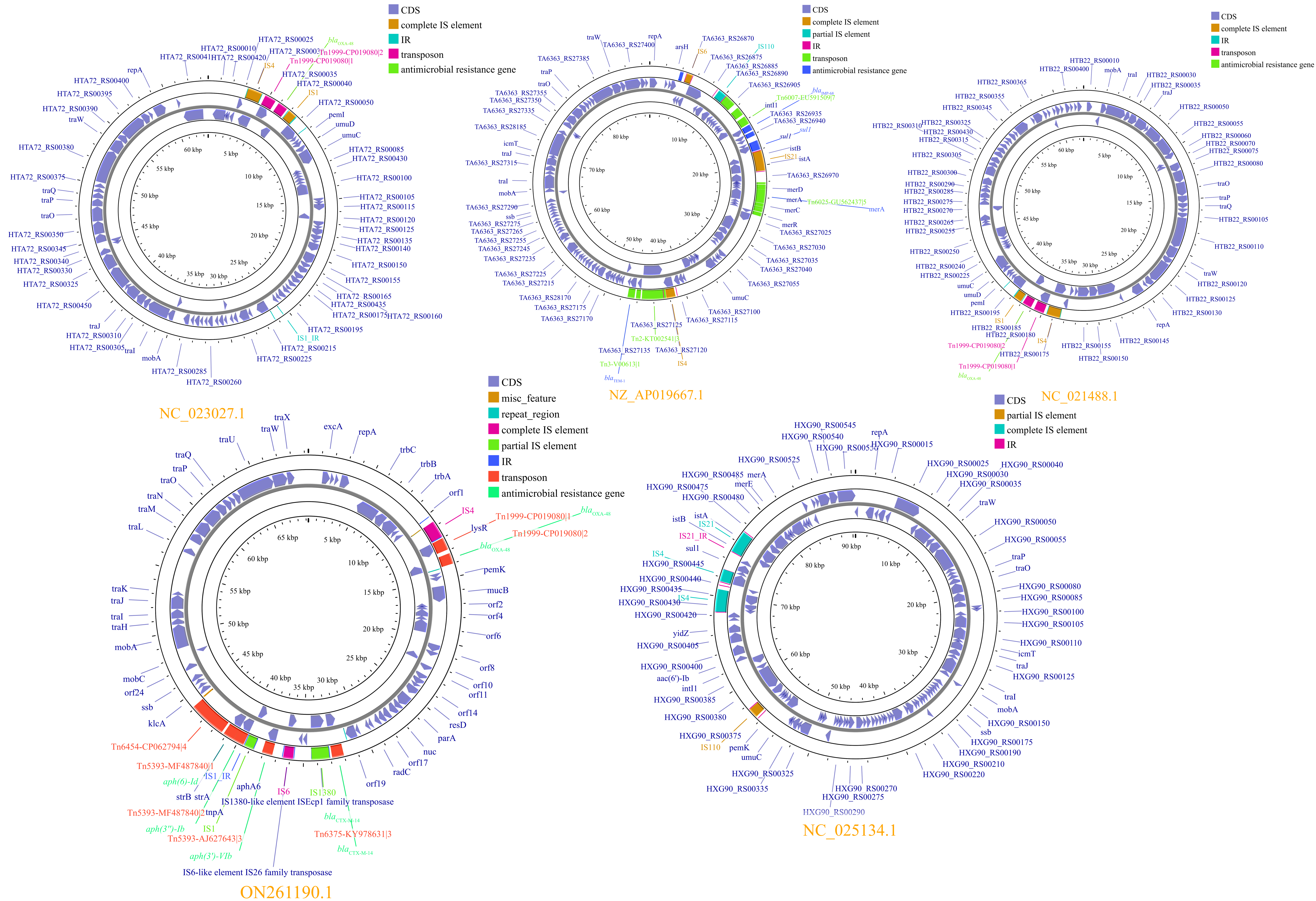

Supplementary figure 5. IncL/M: pE71T, pEGY22_CTX-M-14, pFOX-7a, pKPoxa-48N1, pTMTA63632. The circle diagram is a single plasmid, which plots their resistance genes, genes responsible for binding and the existence of internal mobile genetic elements.

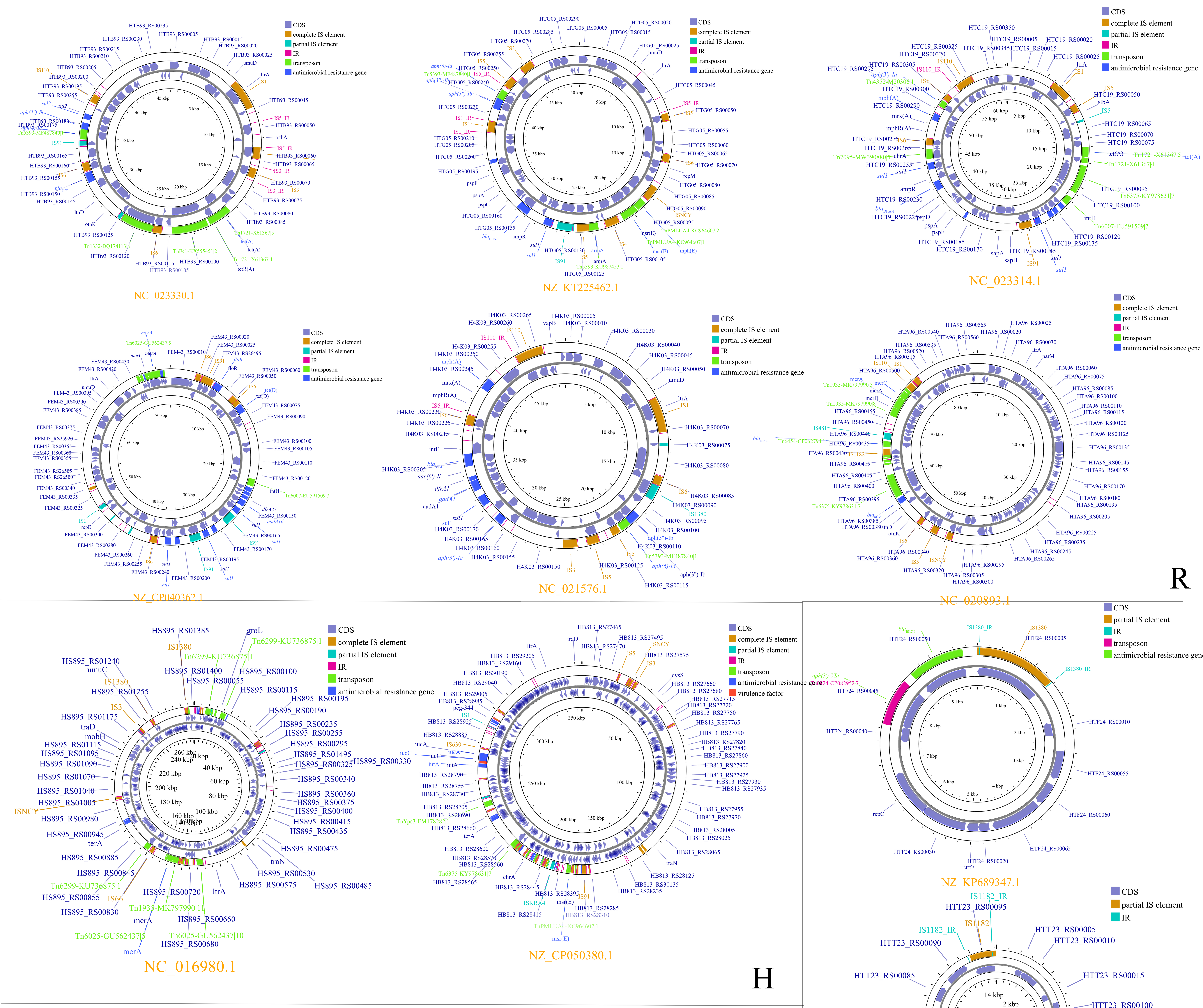

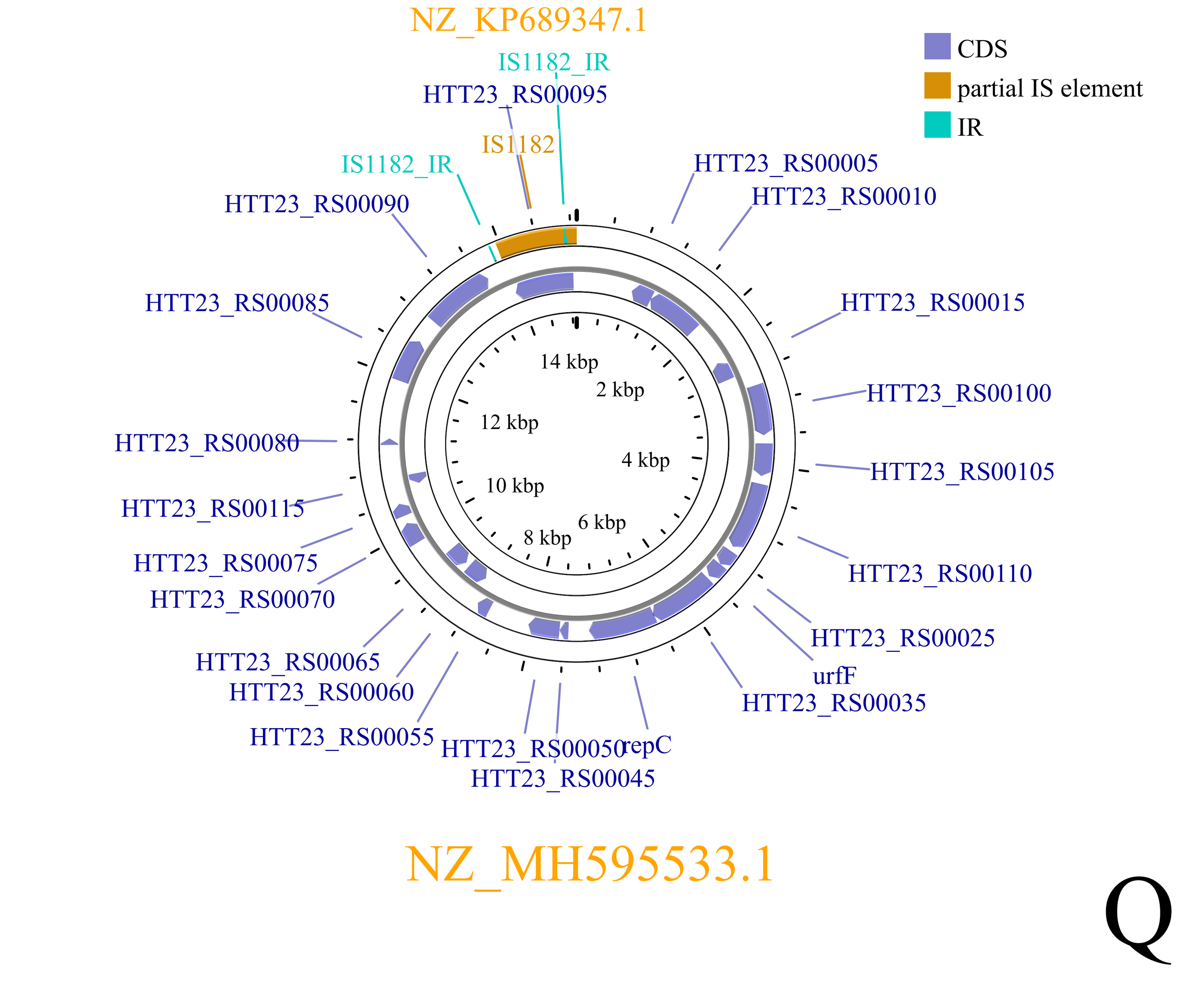

Supplementary figure 6. The other plasmid groups include IncR(A): pKP1780, pKPC-LK30, pKPS30, pKPS77, pR50-74, pYDC676; IncH(B): p51015_NDM-1, pNDM-MAR; IncQ(C): p60136, pKPN535a; IncP(D): pMCR_KP1511.The circle diagram is a single plasmid, which plots their resistance genes, genes responsible for binding and the existence of internal mobile genetic elements.
