# Supplementary material for: Mobile genetic elements encoding antibiotic resistance genes and virulence genes in Klebsiella pneumoniae: important pathways for the acquisition of virulence and resistance
Source: Front Microbiol. 2025 Feb 24;16:1529157. doi: 10.3389/fmicb.2025.1529157 (PMC11891212; doi:10.3389/fmicb.2025.1529157)
Supplement: Supplementary file 2 [file Data_Sheet_1.docx]

**Supplementary Material**

For the plasmids covered herein, the plasmids were mapped by electronic experiments that included three aspects: the regions responsible for splicing, resistance or virulence genes, and other mobile genetic elements contained in the plasmid.

Experimental steps:

1. Based on the plasmid sequence number, Efetch 22.1 was used to download the sequence in fasta format and Bakta 1.9.4 to predict the gene encoded by the plasmid sequence.
2. Based on the presence or absence of relaxase, T4CP and T4SS genes responsible for conjugative, Plascad 1.17 categorized the sequences into conjugative and mobilizable plasmids.
3. AMRFinderPlus 3.12.8 annotates plasmid resistance genes via the AMRFinderPlus core database.
4. IntegronFinder 2.0.5 annotation of integrons.
5. ISEScan 1.7.2.3 annotation of insertion sequences.
6. database construction based on TnCentral transposon sequence tncentral_isfinder.prot.fa, diamond 2.1.9.163 annotated transposon.
7. virulence factor database (VFDB)-based VFDB_setA_pro sequence build database, diamond 2.1.9.163 annotated transposon.
8. GNU Parallel 20240722 to complete batch processing tasks.
9. Combine the integrator, transposon, insertion sequence, virulence and resistance gene annotation information and position on the plasmid to make a gene annotation information table.
10. Input the plasmid sequence number in Proksee to construct the plasmid map, import the gene annotation information table to complete the annotation.
